# NDFNGO: Enhanced Northern Goshawk Optimization Algorithm for Image Segmentation

**DOI:** 10.3390/biomimetics10120837

**Published:** 2025-12-15

**Authors:** Xiajie Zhao, Zuowen Bao, Yu Shao, Na Liang

**Affiliations:** 1 College of Design, Hanyang University, Ansan 15588, Republic of Korea; zhaoxiajie@hanyang.ac.kr; 2 College of Art, Sungkyunkwan University, Seoul 03063, Republic of Korea; baozuowen@g.skku.edu; 3 Academy of Arts & Design, Tsinghua University, Beijing 100084, China; lana-na@mail.tsinghua.edu.cn

**Keywords:** optimization algorithm, nonlinear differential learning strategy, decay factor, fractional-order adaptive learning strategy, image segmentation

## Abstract

The gradual deterioration of fresco pictorial information presents a formidable obstacle for conservators dedicated to protecting humanity’s shared cultural legacy. Currently, scholars in the field of mural conservation predominantly focus on image segmentation techniques as a vital tool for facilitating mural restoration and protection. However, the existing image segmentation methods frequently fall short of delivering optimal segmentation results. To address this issue, this study introduces a novel mural image segmentation approach termed NDFNGO, which integrates a nonlinear differential learning strategy, a decay factor, and a Fractional-order adaptive learning strategy into the Northern Goshawk Optimization (NGO) algorithm to enhance segmentation performance. Firstly, the nonlinear differential learning strategy is incorporated to harness the diversity and adaptability of differential tactics, thereby augmenting the algorithm’s global exploration capabilities and effectively improving its ability to pinpoint optimal segmentation threshold regions. Secondly, drawing on the properties of nonlinear functions, a decay factor is proposed to achieve a more harmonious balance between the exploration and exploitation phases. Finally, by integrating historical individual data, the Fractional-order adaptive learning strategy is employed to reinforce the algorithm’s exploitation capabilities, thereby further refining the quality of image segmentation. Subsequently, the proposed method was evaluated through tests on twelve mural image segmentation tasks. The results indicate that the NDFNGO algorithm achieves victory rates of 95.85%, 97.9%, 97.9%, and 95.8% in terms of the fitness function metric, PSNR metric, SSIM metric, and FSIM metric, respectively. These findings demonstrate the algorithm’s high performance in mural image segmentation, as it retains a significant amount of original image information, thereby underscoring the superiority of the technology proposed in this study for addressing this challenge.

## 1. Introduction

Murals, recognized as an indispensable and pivotal element of global cultural heritage, are imbued with immense cultural and historical significance [1]. Nevertheless, over the course of extended preservation periods, the information encapsulated within mural images undergoes degradation. This degradation presents formidable obstacles to the restoration and long-term conservation efforts aimed at protecting our world’s cultural heritage [2]. At present, researchers and scholars specializing in mural conservation are wholeheartedly committed to the restoration and preservation of murals. They employ a wide array of scientific methodologies, with the overarching goal of ensuring the protection of murals’ values [3]. In the intricate process of restoring images, the identification of degraded details holds paramount importance. Accurately pinpointing these deteriorated elements is crucial for the successful restoration of murals to their original state [4]. Image segmentation technology emerges as a fundamental and indispensable technique in this context. It functions by dividing an image into multiple distinct regions and uses the feature information it contains. This division enables a more straightforward recognition and analysis of detailed image elements, thereby playing a pivotal role in the restoration process [5]. Among the various image segmentation methods currently available, those based on metaheuristic algorithms have attracted considerable attention. These approaches are favored for their straightforward structure, relatively low computational complexity, and wide-ranging applicability across different types of images and scenarios [6].

Metaheuristic algorithms constitute a significant category of algorithms renowned for their straightforwardness and high computational efficiency. These algorithms are predominantly crafted through the emulation of biological behaviors and natural occurrences observed in the real environment. In terms of common classification schemes, metaheuristic algorithms can be broadly categorized into four main types: those inspired by evolutionary processes, those based on swarm intelligence, those drawing inspiration from physics or chemistry, and those rooted in human—related behaviors [7]. Algorithms within the evolution—based category encompass Differential Evolution (DE) [8], which mimics the process of natural selection to optimize solutions; Genetic Algorithm (GA) [9], inspired by the principles of genetics and evolution to search for optimal solutions; and Biogeography—Based Optimization (BBO) [10], which is modeled on the theory of biogeography to solve optimization problems. Among swarm—based metaheuristic algorithms, we have Particle Swarm Optimization (PSO) [11], which simulates the social behavior of bird flocking or fish schooling to find the optimal solution; Slime Mold Algorithm (SMA) [12], inspired by the foraging behavior of slime molds; and Whale Optimization Algorithm (WOA) [13], which is based on the hunting behavior of humpback whales. In addition, there are also the Puma Optimizer [14], the Polar Fox Optimization Algorithm [15], the Electric Eel Foraging Optimization Algorithm [16], and the Elk Herd Optimizer [17]. Physics/chemistry—inspired metaheuristic algorithms include Multi—verse Optimizer (MVO) [18], which is inspired by the multi—verse theory in cosmology; Atom Search Optimization (ASO) [19], modeled on the interaction between atoms; and Water Evaporation Optimization (WEO) [20], which draws inspiration from the water evaporation process. In addition, there exist the Wave Search Algorithm [21], the Convex Combination Search Algorithm [22], and the Nizar Optimization Algorithm [23]. Three notable examples of metaheuristics inspired by human activities include: the Search and Rescue (SAR) method, modeled after emergency operations [24], the Poor and Rich Optimization (PRO) technique, which draws upon socioeconomic dynamics [25], and Teaching-Learning-Based Optimization (TLBO), which emulates educational processes [26]. Due to their high computational efficiency and relatively simple implementation, researchers have put forward a large number of image segmentation algorithms based on metaheuristic algorithms. These algorithms are aimed at improving the overall quality of image segmentation. In addition, there are also the Mother Optimization Algorithm [27] and the Literature Research Optimizer [28].

Over the past few years, scholars have presented a diverse array of image segmentation algorithms grounded in metaheuristic principles to tackle the complex challenges encountered in medical and intricate image analysis scenarios. For example, Lian et al. put forward the Parrot Optimizer (PO), which draws inspiration from the behavioral patterns of African Grey Parrots. The primary objective of PO is to elevate the similarity of segmentation results and enhance the overall quality of images. In the context of disease diagnosis and medical image segmentation tasks, PO has demonstrated robust capabilities in both global and local search processes. This characteristic positions PO as a highly competitive algorithm for improving the outcomes of image segmentation [29]. Motivated by the complexities of medical image segmentation, Yuan et al. presented the Artemisinin Optimization (AO) algorithm as a novel computational method. The AO method effectively balances global exploration and local exploitation. In experiments involving six-threshold segmentation of 15 breast cancer pathology images, AO exhibited excellent optimization capabilities. Its performance surpassed eight leading contemporary methods across a series of metrics, including accuracy, feature similarity, PSNR, and SSIM, highlighting its considerable promise for medical image analysis [30]. The mechanisms of Lévy flight and Cauchy distribution are integrated into a unified framework combining the Lévy-Cauchy Arithmetic Optimization Algorithm (LCAOA) with Rough K-Means (RKM) by Arunita’s research group. These mechanisms work in concert to sustain an optimal equilibrium between global exploration and local exploitation, with the model’s performance being additionally elevated through opposition-based learning. Experimental results across multiple image categories—including color, oral pathology, and leaf images—demonstrated high feature similarity and accuracy, validating the effectiveness of the proposed approach [31]. The Arithmetic Optimization Algorithm–Harris Hawks Optimizer (AOA-HHO), introduced by Qiao’s research group, was engineered for multi-level thresholding segmentation. It achieved top-tier results across all seven tested tiers, excelling specifically in segmentation fidelity, objective function scores, PSNR, and SSIM. It consistently outperformed several benchmark algorithms, confirming its strong effectiveness for multi-level threshold-based image segmentation tasks [32]. Chen et al. tackled the issue of segmentation inefficiencies by introducing the Poplar Optimization Algorithm (POA). In evaluations across six benchmark images, the Pelican Optimization Algorithm (POA) demonstrated exceptional efficacy in multi-threshold segmentation tasks. Its core mechanism centers on augmenting population diversity to refine the search process, thereby offering a more potent solution for image segmentation challenges [33]. Houssein and his colleagues introduced the Snake Optimization algorithm with Opposition—Based Learning (SO—OBL). This algorithm was specifically designed to enhance the segmentation of CT scans for the diagnosis of liver diseases. Through a series of experimental investigations, it was found that SO—OBL exhibits remarkable proficiency in global optimization and multi—level segmentation tasks. When compared with other competing metaheuristic algorithms using metrics such as Feature Similarity Index Measure (FSIM), Structural Similarity Index Measure (SSIM), and Peak Signal—to—Noise Ratio (PSNR), SO—OBL consistently outperformed them. This outstanding performance serves as strong evidence of its high efficiency within computer—aided diagnostic systems [34]. By incorporating opposition-based learning into the Dandelion Optimization Algorithm, Wang et al. developed an enhanced multi-threshold segmentation technique for breast cancer imagery. This strategy enabled more accurate delineation of lesion areas and yielded superior performance metrics, confirming its capability to process the intricate cellular morphology found in such medical images [35]. Aiming to mitigate the Whale Optimization Algorithm’s (WOA) propensity for premature convergence and constrained local search, Wang and colleagues engineered an enhanced variant: the Crossover and Removal of Similarity WOA (CRWOA). Empirical studies involving multi-level thresholding on ten grayscale benchmarks demonstrated that CRWOA achieves not only accelerated convergence velocity but also enhanced segmentation precision relative to five established algorithms, underscoring its performance ascendancy over the standard WOA [36].

In the realm of image processing research, a substantial body of previous studies has carried out in-depth investigations and provided solid evidence to validate the remarkable effectiveness of image segmentation methods that are grounded in metaheuristic algorithms. These studies have also clearly shown that when learning strategies are integrated into these metaheuristic—based approaches, there is a significant enhancement in their overall segmentation performance. Learning strategies can enable the algorithms to better adapt to the characteristics of the images, learn from the data patterns, and thus optimize the segmentation process. However, despite their general effectiveness, the existing metaheuristic approaches are not without flaws. When confronted with domain—specific segmentation challenges, especially in the case of mural image segmentation, these approaches frequently encounter difficulties. Mural images often contain complex and unique features, such as intricate patterns, historical textures, and a wide range of colors. The existing metaheuristic algorithms tend to converge to suboptimal threshold combinations during the segmentation process. This suboptimal convergence leads to a poor retention of the crucial feature information within the mural images. As a consequence, the quality of the segmentation results is severely degraded, with inaccurate boundaries, loss of fine details, and a general inability to accurately represent the original mural content. Given these pressing challenges, it has become an urgent and imperative task in the field of image segmentation research to propose a brand-new optimization algorithm. Fortunately, the Northern Goshawk Optimization (NGO) [37] has been demonstrated to be a robust optimization algorithm with highly efficient search capabilities. Similarly to common optimization algorithms, NGO is a two-stage optimization approach. However, a closer examination of the operational structure of the NGO algorithm reveals a certain simplicity in its programming. This implies that when employing this algorithm to solve optimization problems, it offers advantages such as lower computational costs and higher responsiveness, which precisely align with the requirements for art image segmentation. Moreover, similar research on the NGO algorithm [38,39] has confirmed its excellent applicability and scalability, enabling it to be effectively applied to solve a wide range of optimization problems. Beyond that, studies on the NGO algorithm have also validated its robustness in solving optimization problems, a trait that is particularly crucial for art image segmentation. This investigation employs the NGO algorithm as the baseline method for segmenting mural imagery. Nevertheless, the approach demonstrates three inherent constraints when processing intricate mural features: insufficient global search capacity, inadequate local intensification capability, and poor exploration-exploitation coordination. This triad of limitations adversely affects the algorithmic performance and results in diminished feature retention. To overcome these drawbacks, this research proposes three effective enhancement strategies and presents an improved version of the NGO algorithm, termed NDFNGO. First, to strengthen the global exploration capacity of the original NGO, a nonlinear differential learning strategy is introduced. By leveraging the adaptability and diversity of the differential mechanism, the algorithm achieves more accurate localization of optimal segmentation threshold regions. Second, to mitigate the imbalance between global exploration and local exploitation, a decay factor is incorporated. Through the nonlinear effects of exponential, cosine, and arctangent functions, this factor helps establish a more stable transition between the two search phases. Finally, to enhance the algorithm’s local exploitation capability, a fractional-order adaptive learning strategy is developed that integrates historical individual information. This approach improves fine-tuning performance and enhances the quality of the segmentation threshold combinations. The principal contributions of this study are summarized as follows:

- By leveraging the diversity and adaptability of the differential strategy, a nonlinear differential learning strategy is proposed to enhance the global search capability of the NGO algorithm.
- Leveraging the dynamic behaviors inherent in exponential, cosine, and arctangent functions, a decay factor is incorporated to fine-tune the equilibrium between the NGO algorithm’s global search breadth and local refinement depth.
- By incorporating historical individual information, a Fractional-order adaptive learning strategy is proposed to strengthen the algorithm’s local exploitation ability.
- By integrating the aforementioned three improvement strategies into the NGO algorithm, the NDFNGO algorithm is proposed.
- The utilization of the NDFNGO algorithm for segmenting mural imagery successfully substantiated the viability of this technique, confirming its considerable potential for this specialized field.

The structural organization of this paper proceeds as follows: Section 2 elaborates on the mathematical formulation and operational workflow of the NGO algorithm. Section 3 presents the three improvement strategies and proposes the NDFNGO algorithm. Section 4 primarily focuses on evaluating the performance of NDFNGO in engineering optimization problems. Section 5 primarily applies the NDFNGO algorithm to solve the mural image segmentation problem, confirming its potential as a promising mural segmentation method. Section 6 synthesizes the core findings of this research and delineates prospective avenues for subsequent investigation.

## 2. Mathematical Model of Northern Goshawk Optimization

This section delineates the mathematical framework of the NGO algorithm, a metaheuristic technique modeled after the hunting strategies employed by the northern goshawk. This predatory behavior mainly consists of two stages. The first stage involves the northern goshawk locating its prey from a distance. By simulating this behavioral pattern, the NGO method acquires its global search ability, thereby guiding the optimization process toward regions harboring promising solutions. The second stage occurs after the northern goshawk has regionally located its prey; it then launches a close-range attack to capture the prey. By simulating this stage, the NGO algorithm attains local exploitation capability, which helps the algorithm further enhance optimization accuracy when solving optimization problems. When utilizing the NGO algorithm to solve optimization problems, it is first necessary to initialize a set of individuals with searching ability, namely, the population initialization phase. Subsequently, the initialized population is optimized by simulating the aforementioned global exploration phase and local exploitation phase to achieve higher-quality solutions for optimization problems. The subsequent exposition will provide a detailed breakdown of the NGO algorithm’s computational framework. This encompasses the mathematical formulation of its population initialization, global exploration, and local exploitation stages, ultimately presenting its comprehensive methodology for addressing optimization challenges.

### 2.1. Population Initialization Phase

As a population-driven metaheuristic, the NGO method commences by initializing a set of candidate solutions, each possessing distinct search capabilities. Every candidate embodies a potential answer to the target optimization problem. The algorithm then refines solution quality through successive global exploration and local intensification stages, progressively evolving the population toward the optimum. Mathematically, the initialized candidate set is formulated as per Equation (1).

(1) $$X={\left[\begin{array}{c} X_{1}\\ \vdots \\ X_{i}\\ \vdots \\ X_{N} \end{array}\right]}_{N\times Dim}={\left[\begin{array}{ccccc} x_{1,1} & \cdots & x_{1,j} & \cdots & x_{1,Dim}\\ \vdots & \ddots & \vdots & \ddots & \vdots \\ x_{i,1} & \cdots & x_{i,j} & \cdots & x_{i,Dim}\\ \vdots & \ddots & \vdots & \ddots & \vdots \\ x_{N,1} & \cdots & x_{N,j} & \cdots & x_{N,Dim} \end{array}\right]}_{N\times Dim}$$

where X represents the generated initial population, while $X_{i}$ denotes the information of the ith individual, which is represented as a one-dimensional vector of length Dim. Here, Dim indicates the number of variables (i.e., the dimensionality) of the problem to be solved. N represents the number of individuals in the generated population, and $x_{i,j}$ signifies the information of the jth dimension for the ith individual. When solving optimization problems, it is necessary to iteratively update individuals and retain those with higher-quality information. To evaluate the quality of each individual, the concept of a fitness function value is introduced. Every individual corresponds to a distinct fitness value, which serves as a measure of its performance. When addressing minimization problems, the optimization process seeks lower fitness function values which denote improved individual characteristics. The numerical evaluation for each population member is explicitly formulated in Equation (2).

(2) $$F(X)={\left[\begin{array}{c} F_{1}=F(X_{1})\\ \vdots \\ F_{i}=F(X_{i})\\ \vdots \\ F_{N}=F(X_{N}) \end{array}\right]}_{N\times 1}$$

where $F_{i}$ represents the fitness function value of the ith individual. Following population initialization, the NGO algorithm refines individual quality through successive execution of its global exploration and local exploitation phases. The forthcoming segment provides a detailed breakdown of the computational principles underlying these core stages.

### 2.2. Global Exploration Phase

**Figure 1:**
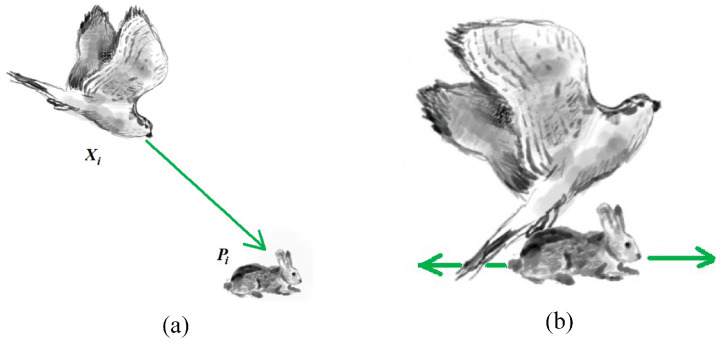
Northern goshawk attack simulation diagram. (**a**) Long range attack. (**b**) Close range attack.

This section’s central objective is the mathematical formulation of the NGO algorithm’s global exploration stage. This phase emulates the northern goshawk’s hunting strategy where it assaults prey by randomly targeting individuals within the prey population. This process is illustrated in Figure 1a. Due to this random prey selection process, individuals can conduct effective searches throughout the population space, thereby achieving the global exploration phase of the algorithm and enhancing its ability to locate the global optimal solution. Specifically, the search method of individuals during the global exploration phase is represented by Equation (3).

(3) $$x_{i,j}^{new}=\left\{\begin{array}{ll} x_{i,j}+r\left(p_{i,j}-I\cdot x_{i,j}\right), & if\;F_{P_{i}}<F_{i}\\ x_{i,j}+r\left(x_{i,j}-p_{i,j}\right), & if\;F_{P_{i}}\geq F_{i} \end{array}\right.$$

The new state for the, jth dimensional of ith individual, after the global exploration phase, is denoted by $x_{i,j}^{new}$. This update utilizes the corresponding dimension $x_{i,j}$ of the current individual, a random number r from the interval [0, 1], and a constant I randomly chosen from {1, 2}. The update also incorporates the jth dimensional $p_{i,j}$ of a randomly selected individual from the population, and $F_{P_{i}}$ represents the fitness function value corresponding to individual $P_{i}$. Notably, $P_{i}$ is selected using Equation (4).

(4) $$P_{i}=X_{k},\;i=1,2,\ldots ,N,\;k=1,2,\ldots ,i-1,i+1,\ldots ,N$$

After the position update through the global exploration phase, Equation (5) will be employed to retain the new states of individuals, thereby achieving the goal of population optimization.

(5) $$X_{i}=\left\{\begin{array}{ll} X_{i}^{new}, & F_{i}^{new}<F_{i}\\ X_{i}, & F_{i}^{new}\geq F_{i} \end{array}\right.$$

where $X_{i}^{new}$ denotes the new state of the ith individual after being updated through the global exploration phase, and $F_{i}^{new}$ represents the fitness function value corresponding to the individual $X_{i}^{new}$. The global exploration stage facilitates the renewal of population members, enabling the algorithm to identify the domain containing the globally optimal solution. Following this, the local refinement process is activated to thoroughly exploit the identified promising area, thus ensuring high convergence accuracy. A comprehensive elaboration of the local search mechanism in the NGO algorithm will be presented in the subsequent section.

### 2.3. Local Exploitation Phase

The mathematical formulation of the local exploitation stage in the NGO algorithm is elaborated in this section. In this phase, the northern goshawk performs close-range assaults on its target to accomplish successful capture, as depicted in Figure 1b. Emulating this behavioral pattern enables the NGO algorithm to substantially improve its refinement of the promising solution area, leading to outstanding optimization accuracy and convergence velocity. Specifically, Equation (6) is employed to represent the position update method for individuals during the local exploitation phase.

(6) $$x_{i,j}^{new}=x_{i,j}+R\cdot (2r-1)\cdot x_{i,j}$$

where $x_{i,j}^{new}$ represents the new state of the jth dimensional information of the ith individual after being updated through the local exploitation phase, while $x_{i,j}$ denotes the jth dimensional information of the ith individual. r signifies a random number within the interval [0, 1], and R indicates the prey attack radius, which is defined by Equation (7).

(7) $$R=0.02\cdot \left(1-\frac{t}{T}\right)$$

where t represents the current iteration number, and T denotes the maximum number of iterations. Subsequently, Equation (5) is employed to retain the state information of individuals during the local exploitation phase. By effectively exploiting the optimal solution region through the local exploitation phase, the algorithm can enhance its convergence accuracy and speed when solving optimization problems, thereby improving its overall optimization performance.

### 2.4. Implementation Framework of NGO

**Figure 2:**
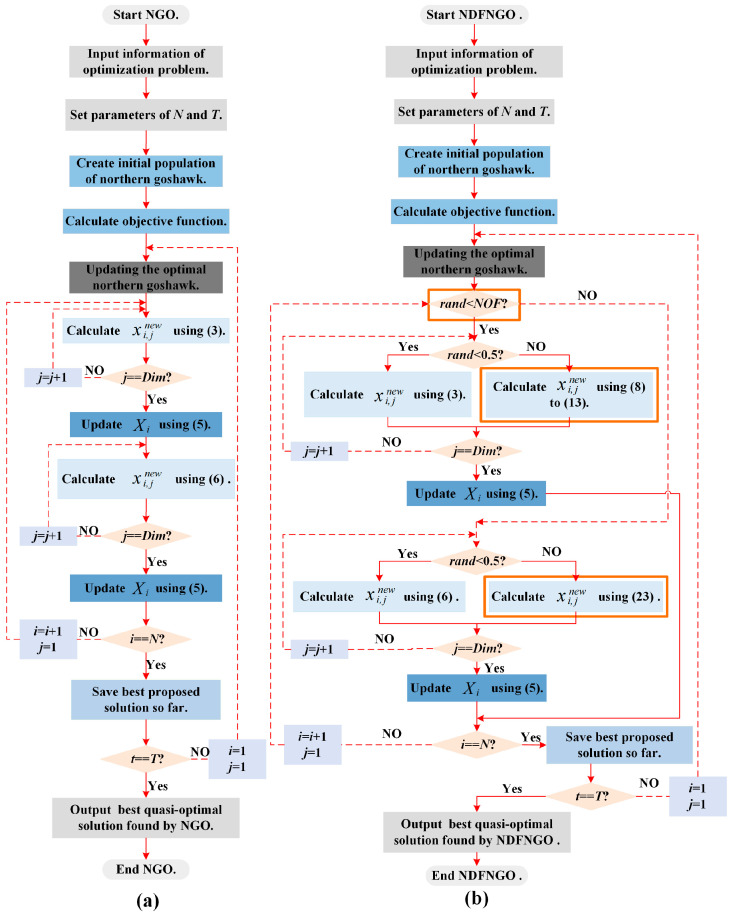
The algorithm flow chart. (**a**) NGO. (**b**) NDFNGO.

Based on the mathematical models previously established for the initialization, global exploration, and local exploitation stages of the NGO algorithm, this section proceeds to synthesize its operational workflow. When tackling real-world optimization challenges, the NGO algorithm first initializes a set of individuals that can represent potential solution schemes for the problem to be optimized, namely, the initialized population. Following this, the quality of these candidate solutions is progressively refined via the algorithm’s global exploration and local refinement mechanisms, enabling the obtained solution schemes to better adapt to solving the optimization problem. The algorithm continues iterating until the predefined maximum number of iterations is reached, at which point it terminates the loop and outputs the optimal individual, representing the optimal solution to the optimization problem, thereby achieving adaptive problem-solving. To provide a more intuitive representation of the algorithm’s execution logic, Figure 2a visualizes the flowchart of the NGO algorithm’s operation.

## 3. Mathematical Model of NDFNGO Algorithm

The original NGO algorithm faces several challenges when applied to image segmentation tasks, including limited global exploration, weak local exploitation, and an imbalance between these two phases. These limitations often cause the algorithm to become trapped in local optima while searching for the best threshold combinations, leading to suboptimal segmentation similarity. To overcome these issues, this section enhances the performance of NGO by incorporating three effective strategies: a nonlinear differential learning strategy, a decay factor mechanism, and a fractional-order adaptive learning strategy. First, to address the insufficient global exploration ability of the original NGO algorithm—which restricts its capacity to effectively identify optimal segmentation threshold regions—a nonlinear differential learning strategy is introduced. This approach integrates the advantages of six differential learning methods with a nonlinear adjustment factor, enhancing both the diversity and adaptability of the algorithm. Consequently, the global search capability of the algorithm is strengthened, improving its efficiency in locating the optimal segmentation threshold region. Second, to resolve the imbalance between global exploration and local exploitation in the original NGO algorithm, a decay factor is proposed. By introducing nonlinear effects derived from exponential, cosine, and arctangent functions, this mechanism achieves a more harmonious transition between exploration and exploitation. This balance allows the algorithm to better escape local optima during the search for the optimal threshold. Finally, to enhance the algorithm’s local exploitation ability, a fractional-order adaptive learning strategy is designed. By incorporating the memory characteristics of fractional-order theory and adapting to historical information differences, this method effectively strengthens the algorithm’s capacity for local fine-tuning, thereby improving the overall quality of the segmented threshold combinations. By integrating these three strategies, an improved algorithm—termed NDFNGO—is proposed. NDFNGO addresses the inherent shortcomings of the original NGO from multiple perspectives, resulting in enhanced structural similarity and improved performance in image segmentation tasks. The subsequent section provides a detailed explanation of the mathematical models of the three learning strategies, along with the operational logic of the NDFNGO algorithm.

### 3.1. Nonlinear Differential Learning Strategy

The original NGO algorithm exhibits a deficiency in global exploration capability when addressing image segmentation problems, resulting in its inability to effectively locate the optimal segmentation threshold region. Therefore, there is an urgent need to propose an efficient global exploration strategy to enhance the algorithm’s capability in locating optimal regions. Xu [40] and Li [41], among others, have demonstrated in their research that differential strategies can effectively enhance the algorithm’s global exploration capability by incorporating information differences among individuals. Inspired by this, we have adopted a differential strategy in NGO to enhance its global exploration performance. Meanwhile, the learning degree of the original differential strategy primarily relies on fixed parameters, which can lead to a significant loss in optimization accuracy despite the enhanced global exploration performance during the algorithm’s evolutionary process. Therefore, we have modified the fixed learning parameters of the original differential strategy, making them adaptive to better suit the nature of solving image segmentation problems. Furthermore, to ensure the diversity of the differential strategies, this section introduces six fundamental differential strategies, which are mainly categorized into global exploration types and local exploitation types. Thus, to better integrate these six differential strategies with the NGO algorithm and enhance its effective global exploration capability, this section also draws on the idea mentioned in reference [42] that nonlinear factors can better control the algorithm’s coordination during the iterative process. We propose a novel nonlinear factor to coordinate these six differential strategies, thereby further enhancing the algorithm’s global exploration performance and improving its ability to locate the globally optimal image segmentation region. Based on the aforementioned ideas, this section proposes a nonlinear differential learning strategy to enhance its global exploration capability. Below, we will introduce the mathematical model of this strategy in detail.

**Figure 3:**
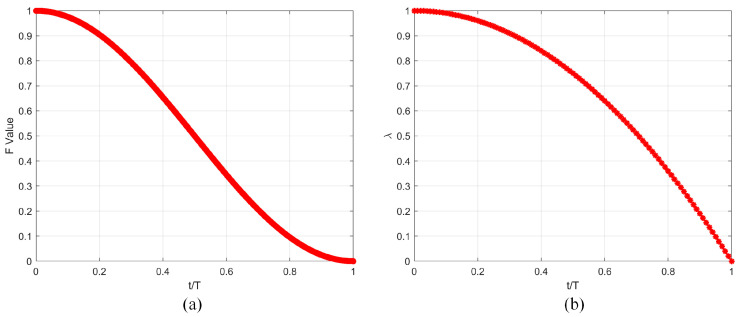
Nonlinear factor variation diagram. (**a**) Equation (14). (**b**) Equation (15).

This strategy primarily integrates six common differential strategies (DE) while considering their enhancing characteristics on the algorithm’s global exploration capability. By combining these differential strategies with a nonlinear factor, the strategy effectively enhances the algorithm’s global exploration capability. Specifically, it encompasses six forms of differential strategies: DE/rand/1, DE/best/1, DE/rand/2, DE/best/2, DE/current-to-rand/1, and DE/current-to-best/1, which are represented by Equations (8) through (13), respectively.

(8) $$X_{i}^{new}=X_{r1}+F\cdot (X_{r2}-X_{r3})$$

(9) $$X_{i}^{new}=X_{best}+F\cdot (X_{r1}-X_{r2})$$

(10) $$X_{i}^{new}=X_{r1}+F\cdot (X_{r2}-X_{r3})+\lambda \cdot (X_{r4}-X_{r5})$$

(11) $$X_{i}^{new}=X_{best}+F\cdot (X_{r1}-X_{r2})+\lambda \cdot (X_{r3}-X_{r4})$$

(12) $$X_{i}^{new}=X_{i}+F\cdot (X_{r1}-X_{i})+\lambda \cdot (X_{r2}-X_{r3})$$

(13) $$X_{i}^{new}=X_{i}+F\cdot (X_{best}-X_{i})+\lambda \cdot (X_{r1}-X_{r2})$$

where $X_{r1}$, $X_{r2}$, $X_{r3}$, $X_{r4}$, and $X_{r5}$ respectively represent the information of distinct individuals within the population, while $X_{best}$ denotes the information of the current best individual in the population. F and λ represent adaptive factors used to adjust the learning degree of the differential strategies. These are expressed using Equations (14) and (15), respectively. Their functional graphs are represented using Figure 3a and Figure 3b, respectively.

(14) $$F=(1+\cos(\pi \cdot (\frac{t}{T})))/2$$

(15) $$\lambda =1-{\left(\frac{t}{T}\right)}^{2}$$

**Figure 4:**
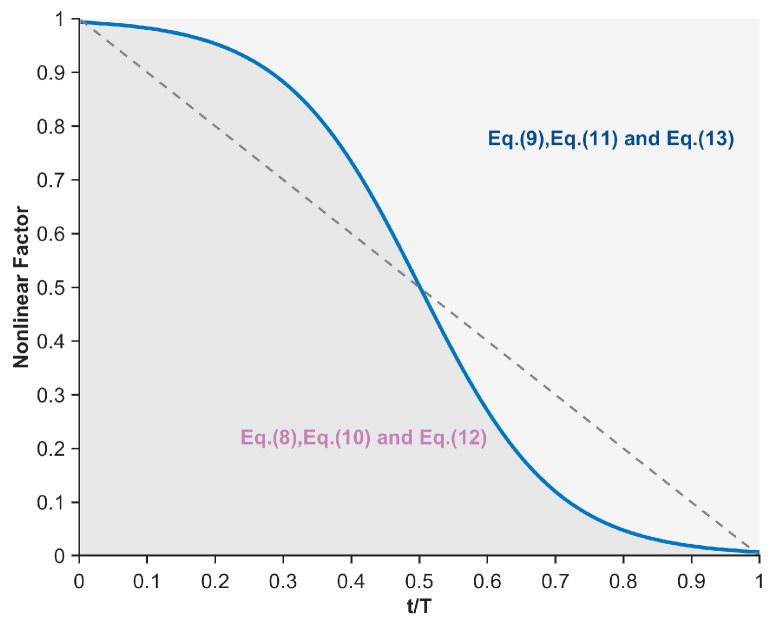
The nonlinear factor corresponding to Equation (16).

In the nonlinear differential learning strategy, we take into account that different forms of differential learning exhibit distinct global exploration characteristics, leading to varying impacts on algorithm performance. Specifically, the differential learning forms DE/rand/1, DE/rand/2, and DE/current-to-rand/1 employ an unconstrained free search mode, where individual mutations are based on random selection. This approach enables coverage of a broader search space, maximizes population diversity, and consequently enhances the probability of locating the global optimum region. Therefore, during the early iterations, these three differential schemes are randomly selected to effectively bolster the algorithm’s global exploration capability. As iterations progress, the extensive search range of DE/rand/1, DE/rand/2, and DE/current-to-rand/1 becomes counterproductive in maintaining the algorithm’s optimization direction. In contrast, differential learning forms such as DE/best/1, DE/best/2, and DE/current-to-best/1 introduce the optimal individual to guide the search direction. The generation of mutant individuals is constrained by the optimal solution, and the search is conducted around it, significantly accelerating the algorithm’s convergence rate and enhancing its ability to trend towards the optimal solution. Hence, during the later iterations, these three differential strategies are randomly selected to primarily reinforce the algorithm’s global exploration capability. This approach ensures both global exploration and the maintenance of the optimization direction, thereby further improving the algorithm’s image segmentation performance. The controlled utilization of these two categories of differential strategies is governed by a nonlinear factor, as depicted in Equation (16), with its graphical representation shown in Figure 4.

(16) $$NF=1-\frac{1}{1+\exp(-k\cdot (t/T-0.5))}$$

where exp(⋅) denotes the exponential operation, and NF represents the nonlinear factor used to control the two types of differential strategies. In this section, the value of k is set to 10. As can be observed from Figure 4, during the early iterations, unconstrained differential learning strategies are primarily employed to maximize the algorithm’s global exploration capability and enhance its ability to locate the optimal segmentation threshold region. In the later iterations, differential learning strategies constrained by the optimal solution are mainly adopted to ensure the determination of precise segmentation thresholds while also maintaining a certain level of global exploration capability. In conclusion, the integration of a nonlinear differential learning strategy substantially strengthens the algorithm’s global search performance. This enhancement directly translates into improved accuracy in identifying the optimal segmentation threshold domain when addressing image segmentation tasks.

### 3.2. Decay Factor

In tackling image segmentation tasks, the standard NGO algorithm demonstrates a pronounced disparity between its global search and localized refinement processes. This structural imbalance frequently leads to premature convergence on suboptimal solutions during the quest for ideal segmentation parameters, consequently diminishing segmentation efficacy. Fortunately, Wang et al. [43] demonstrated in their work that incorporating a nonlinear process between the global exploration phase and the local exploitation phase can effectively enhance the algorithm’s execution balance. Inspired by this, this section aims to propose a nonlinear factor to balance these 2 phases. Additionally, although the nonlinear factor proposed by Wang et al. has somewhat enhanced the algorithm’s balance, it still has certain drawbacks. The main issue lies in its inclusion of an inverse cosine function term, which heavily biases the process toward the global exploration phase. Consequently, the resulting nonlinear factor, to a certain extent, leans more toward the global exploration phase, which may compromise the algorithm’s convergence accuracy. In summary, inspired by this work and addressing its existing issues, this section proposes an enhanced decay factor to better achieve a reasonable balance between the global exploration and local exploitation phases. The decay factor primarily consists of three components: an exponential decay coefficient, a cosine fluctuation coefficient, and an arctangent steepness coefficient. Among them, the exponential decay coefficient and the arctangent steepness coefficient effectively enhance the algorithm’s local exploitation phase, compensating for the deficiencies in Wang et al.’s work. The cosine fluctuation coefficient effectively strengthens the algorithm’s global exploration phase. The combination of these three components enables the proposed decay factor to effectively balance the algorithm’s execution. Specifically, the exponential decay coefficient mainly controls the adaptive decay behavior of the decay factor, the cosine fluctuation coefficient introduces certain variability adjustments, and the arctangent steepness coefficient controls its slope. In detail, the exponential decay coefficient, cosine fluctuation coefficient, and arctangent steepness coefficient are represented by Equations (17) through (19), respectively.

(17) $$Coe_{exp}=\exp(-\lambda \cdot (\frac{t}{T}))$$

(18) $$Coe_{\cos}=\cos\left(\frac{\pi }{2}\cdot {\left(\frac{t}{T}\right)}^{\alpha }\right)$$

(19) $$Coe_{arc}=1-\frac{2}{\pi }\arctan\left(\beta \cdot \left(\frac{t}{T}\right)\right)$$

**Figure 5:**
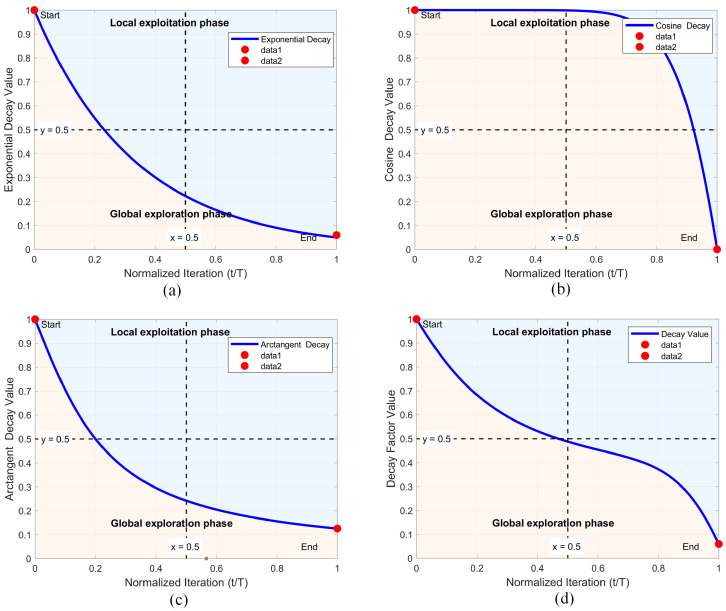
Decay factor. (**a**) Equation (17). (**b**) Equation (18). (**c**) Equation (19). (**d**) Equation (20).

Here, $Coe_{exp}$ represents the exponential decay coefficient, $Coe_{\cos}$ denotes the cosine fluctuation coefficient, and $Coe_{arc}$ signifies the arctangent steepness coefficient. Meanwhile, λ, α, and β are the control parameters for the exponential decay coefficient, cosine fluctuation coefficient, and arctangent steepness coefficient, respectively. Through testing, it has been determined that optimal balancing is achieved when λ = 3, α = 5, and β = 5. The functional graphs of $Coe_{exp}$, $Coe_{\cos}$, and $Coe_{arc}$ with respect to the iteration number t are illustrated in Figure 5a, Figure 5b, and Figure 5c, respectively. As can be observed from the figures, the exponential decay coefficient and the arctangent steepness coefficient predominantly favor the local exploitation phase, whereas the cosine fluctuation coefficient mainly supports the global exploration phase. Therefore, by combining these three coefficients, we propose the decay factor in this section, represented by Equation (20), with its graphical depiction shown in Figure 5d.

(20) $$NOF=\frac{Coe_{exp}+Coe_{\cos}+Coe_{arc}}{3}$$

where NOF denotes the decay coefficient proposed in this work. As illustrated in Figure 5d, the coordinated action of three control parameters guides the algorithm to prioritize global search in early iterations while shifting focus to localized refinement in later stages. This design preserves residual global exploration capacity throughout the process, enabling escape from suboptimal segmentation threshold configurations. Collectively, the introduced decay mechanism successfully creates a dynamic equilibrium between exploration and exploitation in the NGO framework, ultimately enhancing its segmentation accuracy and robustness.

### 3.3. Fractional-Order Adaptive Learning Strategy

**Figure 6:**
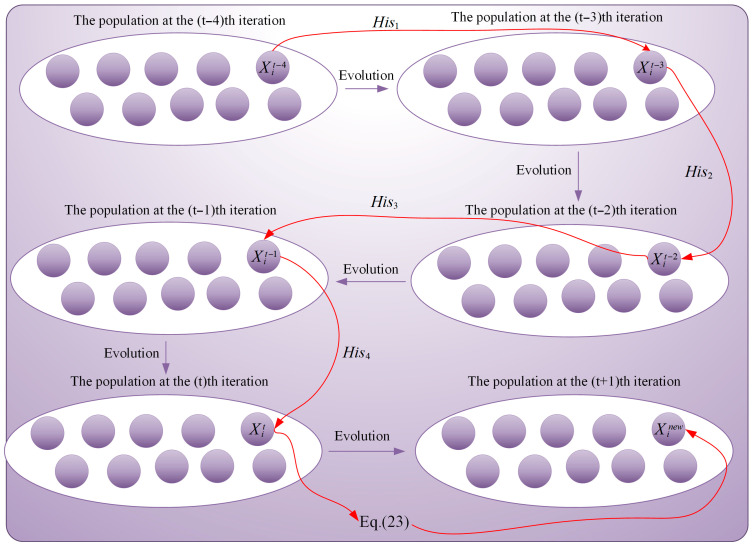
Fractional-order adaptive learning strategy plot.

The conventional NGO methodology exhibits limited refinement capacity in image segmentation contexts, leading to inadequate development of promising threshold regions discovered during global search operations. This deficiency reduces the similarity and quality of the segmented images. Therefore, to alleviate this issue, this section aims to propose an effective exploitation strategy to enhance the algorithm’s local exploitation performance. Seyed et al. [44] pointed out in the literature that leveraging individual historical information through fractional-order theory can effectively strengthen the algorithm’s local exploitation capability. Inspired by this, to effectively enhance the algorithm’s local exploitation capability, this section introduces fractional-order theory into the individual weighting process. However, it is noteworthy that traditional fractional-order learning strategies aim to learn from the discrepancies in historical individual information but fail to consider the learnability of these discrepancies. This can lead to an increased risk of the algorithm falling into local optima traps despite the enhanced local exploitation capability. Therefore, we must take into account the learnability of historical individual discrepancies. Fortunately, Wang et al. proposed a quantitative scheme for discrepancy learnability in the literature [43]. Building on this idea and addressing the shortcomings of traditional fractional-order learning strategies, this section proposes a fractional-order adaptive learning strategy that incorporates an advanced discrepancy learnability measurement scheme to enhance the algorithm’s local mining capability. In the proposed strategy, by leveraging the memory characteristics of fractional-order theory for historical individuals and considering the learnability of each piece of historical information, the algorithm’s local utilization capability is adaptively improved. Specifically, this section utilizes fractional-order theory to learn from the information of an individual’s previous four generations, with these four sets of historical discrepancies represented by Equation (21).

(21) $$\left\{\begin{array}{l} His_{1}=X_{i}^{t}-X_{i}^{t-1}\\ His_{2}=X_{i}^{t-1}-X_{i}^{t-2}\\ His_{3}=X_{i}^{t-2}-X_{i}^{t-3}\\ His_{4}=X_{i}^{t-3}-X_{i}^{t-4} \end{array}\right.$$

where $X_{i}^{t}$ denotes the positional information of the ith individual at the tth iteration, $X_{i}^{t-1}$ represents the positional information of the ith individual at the (t−1)th iteration, $X_{i}^{t-2}$ indicates the positional information of the ith individual at the (t−2)th iteration, $X_{i}^{t-3}$ signifies the positional information of the ith individual at the (t−3)th iteration, and $X_{i}^{t-4}$ denotes the positional information of the ith individual at the (t−4)th iteration. Furthermore, $His_{1}$ represents the information gap between individual $X_{i}^{t}$ and individual $X_{i}^{t-1}$, $His_{2}$ denotes the information gap between individual $X_{i}^{t-1}$ and individual $X_{i}^{t-2}$, $His_{3}$ indicates the information gap between individual $X_{i}^{t-2}$ and individual $X_{i}^{t-3}$, and $His_{4}$ signifies the information gap between individual $X_{i}^{t-3}$ and individual $X_{i}^{t-4}$. Subsequently, by considering the distinctiveness of each difference, the learnability of each gap is calculated, with the learnability of each gap represented by Equation (22).

(22) $$LEA_{k}=\frac{\left\|His_{k}\right\|}{\sum_{k=1}^{4}\left\|His_{k}\right\|},\;\;\;\;(k=1,2,3,4)$$

where $LEA_{k}$ represents the learnability of the kth group of historical information gaps, and “‖⋅‖” denotes the modulus operation. Subsequently, the historical difference information is learned utilizing fractional-order theory, as expressed by Equation (23). This learning process is illustrated in Figure 6.

(23) $$\begin{array}{c} X_{i}^{new}=X_{i}^{t}+\frac{1}{1!}\cdot q\cdot LEA_{1}\cdot His_{1}+X_{i}^{t}+\frac{1}{2!}\cdot q\cdot (1-q)\cdot LEA_{2}\cdot His_{2}+\frac{1}{3!}\cdot q\\ \;\;\;\;\;\;\;\;\;\;\;\;\;\;\;\;\;\;\;\;\;\;\cdot (1-q)\cdot (2-q)\cdot LEA_{3}\cdot His_{3}+\frac{1}{4!}\cdot q\cdot (1-q)\cdot (2-q)\cdot (3-q)\cdot LEA_{4}\cdot His_{4} \end{array}$$

where q represents an adaptive factor, as defined by Equation (24), which is employed to dynamically regulate the extent of learning from historical individual information. By updating individuals through the Fractional-order adaptive learning strategy, the algorithm effectively leverages the learnability of historical information to enhance its local exploitation capability. This, in turn, improves the algorithm’s performance in solving image segmentation problems, achieving higher-quality image segmentation results.

(24) $$q=\frac{1}{1+e^{\left(t/T\right)}}\cdot \cos(2\cdot \pi \cdot (\frac{t}{T}))$$

### 3.4. Implementation Framework of NDFNGO

To overcome the limitations of the standard NGO approach in image processing applications, we introduce an augmented framework designated NDFNGO. This enhanced architecture incorporates three synergistic components: a nonlinear differential learning mechanism, a dynamic decay coefficient, and a fractional-order adaptive learning module, collectively engineered to elevate segmentation efficacy. The mathematical models of these three learning strategies have been elaborated upon in the preceding subsections. In this section, we will delineate the operational logic of NDFNGO when executing optimization problems, to provide an intuitive representation of NDFNGO’s operational logic, Figure 2b displays the flowchart of the algorithm, with the improvement points highlighted by brown rectangles.

### 3.5. Time Complexity Analysis

This subsection focuses on computational complexity analysis of the proposed NDFNGO algorithm. The foundational NGO framework operates through three sequential procedures: solution set initialization, worldwide search, and regional refinement. Throughout these stages, fitness evaluation constitutes the primary computational overhead. Therefore, the time complexity of the population initialization phase is O(N), where N represents the population size. The time complexity of the global exploration phase is O(T⋅N⋅Dim), where T denotes the maximum number of algorithm iterations and Dim signifies the dimensionality of the optimization problem. Similarly, the time complexity of the local exploitation phase is also O(T⋅N⋅Dim). Consequently, the overall time complexity of the NGO algorithm when executing optimization tasks is O(N⋅(1+2⋅T⋅Dim)). Similarly, the NDFNGO algorithm follows a similar execution logic as the NGO algorithm. The enhancement solely incorporates a probabilistically balanced update mechanism across both exploration and development stages, incurring no supplementary computational burden per iteration cycle. It should be emphasized, however, that the introduced decay coefficient dynamically regulates the transition between these phases, yielding an overall iterative complexity of O(T⋅N⋅Dim). Consequently, the comprehensive computational burden encompassing initialization remains O(N⋅(1+T⋅Dim)).

## 4. Experimental Validation and Discussion on Real-World Applications

This subsection conducts comprehensive performance evaluation of the NDFNGO methodology using practical engineering design challenges. The assessment employs four established benchmark problems: Tension/Compression Spring Design (TCSD), Welded Beam Design (WBD), 10 bar Truss Design (TenBTD), and Three-bar Truss Design (TBTDP) [45]. All trials were executed through 30 independent runs with randomized initializations. Statistical performance measures, including mean fitness values and corresponding standard deviations derived from these repeated experiments, are summarized in Table 1. From the table, it can be observed that, for the TCSD case problem, the NDFNGO algorithm and the ANBPO algorithm both achieved an optimized fitness value of 0.0127, securing the top position. In terms of the standard deviation metric, the NDFNGO algorithm demonstrated superiority, indicating better solution stability. For the WBD problem, the NDFNGO, NGO, and ANBPO algorithms all achieved an optimized fitness value of 1.6702, tying for first place, with the NDFNGO algorithm showing a certain advantage in the standard deviation metric. For the TenBTD problem, the NDFNGO algorithm attained an optimized fitness value of 524.4800, ranking first, while also demonstrating better stability in the standard deviation metric. Additionally, for the TBTDP problem, the NDFNGO algorithm continued to exhibit a strong solution advantage in terms of the fitness function value. In summary, through an analysis of the experimental results on engineering optimization problems, it is confirmed that the NDFNGO algorithm proposed in this paper possesses favorable global optimization performance and exhibits certain advantages in solving real-world engineering optimization problems.

## 5. Experimental Results and Discussion on Image Segmentation

**Figure 7:**
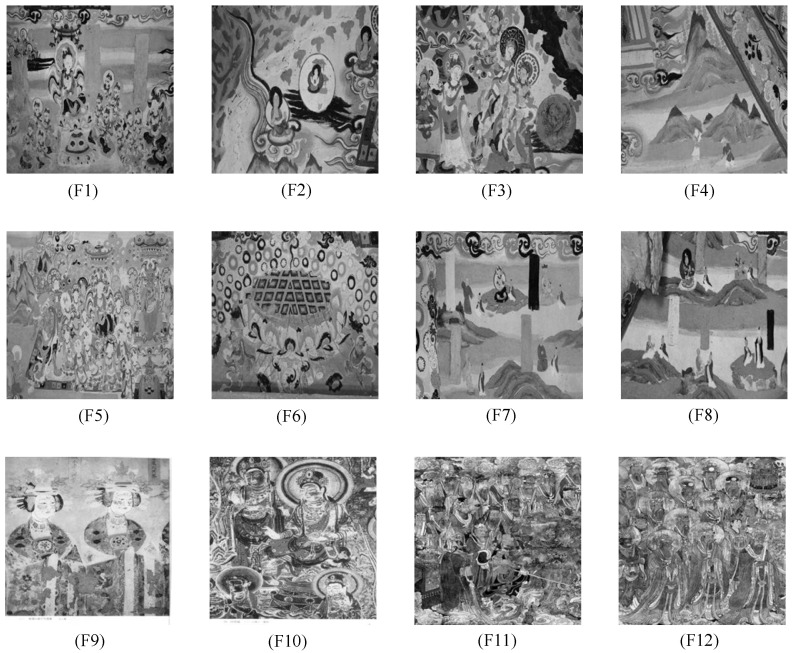
Original mural image.

This section assesses the segmentation efficacy of the proposed NDFNGO approach when applied to mural imagery. The validation employs twelve distinct mural samples, with detailed visual characteristics and specifications provided in Figure 7. Among them, the twelve mural images involved in this experiment are sourced from a standard dataset for mural image processing. All images in this dataset have undergone image standardization procedures. Moreover, it encompasses a diverse range of mural types and is thus extensively utilized for performance testing of mural image segmentation algorithms. This dataset can be accessed via the link: https://ww2.mathworks.cn/matlabcentral/fileexchange/181489-mural-image-segmentation-dataset (accessed on 1 December 2025). The twelve images selected for this study primarily feature three typical mural styles: figures, architecture, and landscapes. This curated dataset ensures thorough assessment of algorithmic capabilities. For visual comparison of segmentation outcomes, the proposed NDFNGO method is evaluated against five established high-performance algorithms, whose parameter configurations are detailed in Table 2. To ensure statistical reliability, all experiments underwent 30 independent trials with randomized initializations, with reported results representing averaged performance. The experimental framework maintained uniform conditions with maximum iterations fixed at 100 and population size set at 40. Comprehensive evaluation encompassed multiple dimensions: population diversity analysis, exploration-exploitation equilibrium assessment, strategy effectiveness verification, fitness value evolution, Friedman non-parametric testing, and quantitative metrics including PSNR, SSIM, FSIM, alongside overall performance ranking.

Meanwhile, in the mural image segmentation experiment, we set the number of segmentation thresholds to four values: 2, 4, 6, and 8. Our selection of these specific threshold numbers is primarily based on the research studies on image segmentation conducted by Houssein [46] and Mohamed [47]. They pointed out that although a larger number of segmentation thresholds can enhance the level of image subdivision, the computational cost also escalates exponentially as the number of thresholds increases. In contrast, it has been demonstrated that choosing 2, 4, 6, and 8 as the number of segmentation thresholds can effectively segment and extract image features. This approach not only reduces computational costs but also effectively ensures the quality of image segmentation. Moreover, if the number of image segmentation thresholds is excessively large, the final segmentation results of the image are significantly susceptible to the influence of noise points in the picture, which is detrimental to the extraction of key image information. Therefore, it is reasonable to opt for 2, 4, 6, and 8 as the number of segmentation thresholds.

**Table 2:** Compare algorithm parameter settings.

Algorithms	Year	Parameter Settings
OPBNGO [48]	2025	$R=0.02\cdot \left(1-\frac{t}{T}\right)$
NGO [37]	2021	$R=0.02\left(1-\frac{t}{T}\right)$
ANBPO [43]	2025	$NF_{arccos}=\arccos\left(\frac{t}{Max_{iter}}\right)/\left(\frac{\pi }{2}\right)$
DENGO [49]	2024	$R=0.02\left(1-\frac{t}{T}\right)$
IMODE [50]	2020	D=2,Arch_rate=2.6

### 5.1. Model of Mural Image Segmentation

This section formulates the computational framework for mural image segmentation. The methodology adopts the Otsu technique, which operates by determining an optimal threshold that maximizes inter-class variance among segmented regions. The proposed NDFNGO algorithm serves as the search mechanism for identifying this optimal threshold configuration. The objective function is designed to maximize inter-class variance in the segmented output. Through exhaustive exploration of the threshold solution space, the method identifies the most effective segmentation parameters to ensure high-quality mural image classification. The subsequent subsection provides detailed specifications of the fitness function model. Firstly, assume that the grayscale pixel matrix of the image to be segmented is denoted as I, and the image comprises L grayscale levels. Let $n_{i}$ represent the number of pixels with a grayscale level of i. Based on the aforementioned definitions, the total number of pixels N in image I can be expressed by Equation (25).

(25) $$N=\sum_{i=0}^{L-1}n_{i}$$

Subsequently, Equation (26) is employed to calculate the proportion of pixels with a grayscale level of i in the entire image I.

(26) $$P_{i}=\frac{n_{i}}{N},\;\;\;\;\;\;i=0,1,\ldots ,L-1$$

Given an image segmentation threshold t, the input image partitions into two distinct regions: pixels with intensity values within [0, t] constitute the foreground, while those within [t + 1, L − 1] form the background, where L represents the maximum intensity level. Defining ω_0_ as the foreground pixel ratio, μ_0_ as its mean intensity, ω_1_ as the background ratio, μ_1_ as its mean intensity, μ as the global mean intensity, and σ^2^ as the inter-class variance, these parameters are mathematically derived through Equations (27)–(32).

(27) $$\omega_{0}=\sum_{i=0}^{t}P_{i}$$

(28) $$\mu_{0}=\sum_{i=0}^{t}\frac{iP_{i}}{\omega_{0}}$$

(29) $$\omega_{1}=\sum_{i=t+1}^{L-1}P_{i}$$

(30) $$\mu_{1}=\sum_{i=t+1}^{L-1}\frac{iP_{i}}{\omega_{1}}$$

(31) $$\mu =\sum_{i=0}^{k-1}\omega_{i}\mu_{i}=\sum_{i=0}^{L-1}iP_{i}$$

(32) $$\nu (t)=\omega_{0}{\left(\mu_{0}-\mu \right)}^{2}+\omega_{1}{\left(\mu_{1}-\mu \right)}^{2}=\omega_{0}\omega_{1}{\left(\mu_{0}-\mu_{1}\right)}^{2}$$

Subsequently, the optimal segmentation threshold $t_{best}$ is selected in accordance with the definition provided in Equation (33).

(33) $$t_{best}=\arg\;\max_{0\leq t\leq L}\nu (t)$$

For multi-threshold segmentation with k thresholds, the inter-class variance υ is generalized as expressed in Equation (34).

(34) $$\begin{array}{cc} \nu (t_{1},t_{2},\ldots ,t_{k}) & =\omega_{0}\omega_{1}{\left(\mu_{0}-\mu_{1}\right)}^{2}+\omega_{0}\omega_{2}{\left(\mu_{0}-\mu_{2}\right)}^{2}+\cdots \\ & +\omega_{0}\omega_{k}{\left(\mu_{0}-\mu_{k}\right)}^{2}+\omega_{1}\omega_{2}{\left(\mu_{1}-\mu_{2}\right)}^{2}+\cdots \\ & +\omega_{1}\omega_{3}{\left(\mu_{1}-\mu_{3}\right)}^{2}+\cdots +\omega_{k-1}\omega_{k}{\left(\mu_{k-1}-\mu_{k}\right)}^{2} \end{array}$$

Here, the calculation methods for $\omega_{i}$ and $\mu_{i}$ are extended to Equations (35) and (36), respectively.

(35) $$\omega_{i-1}=\sum_{i=t_{i-1}+1}^{t_{i}}P_{i},1\leq i\leq k+1$$

(36) $$\mu_{i-1}=\sum_{i=t_{i-1}+1}^{t_{i}}\frac{iP_{i}}{\omega_{i-1}},\;1\leq i\leq k+1$$

The optimal combination of image segmentation thresholds, denoted as $T_{best}=(t_{1},\ldots ,t_{k})$, is determined according to the rule defined in Equation (37).

(37) $$T_{best}=\arg\;\max_{0\leq t_{1}\leq t_{2}\leq \cdots \leq t_{k}}\;\;v(t_{1},t_{2},\ldots ,t_{k})$$

### 5.2. Decay Factor Parameter Settings

**Figure 8:**
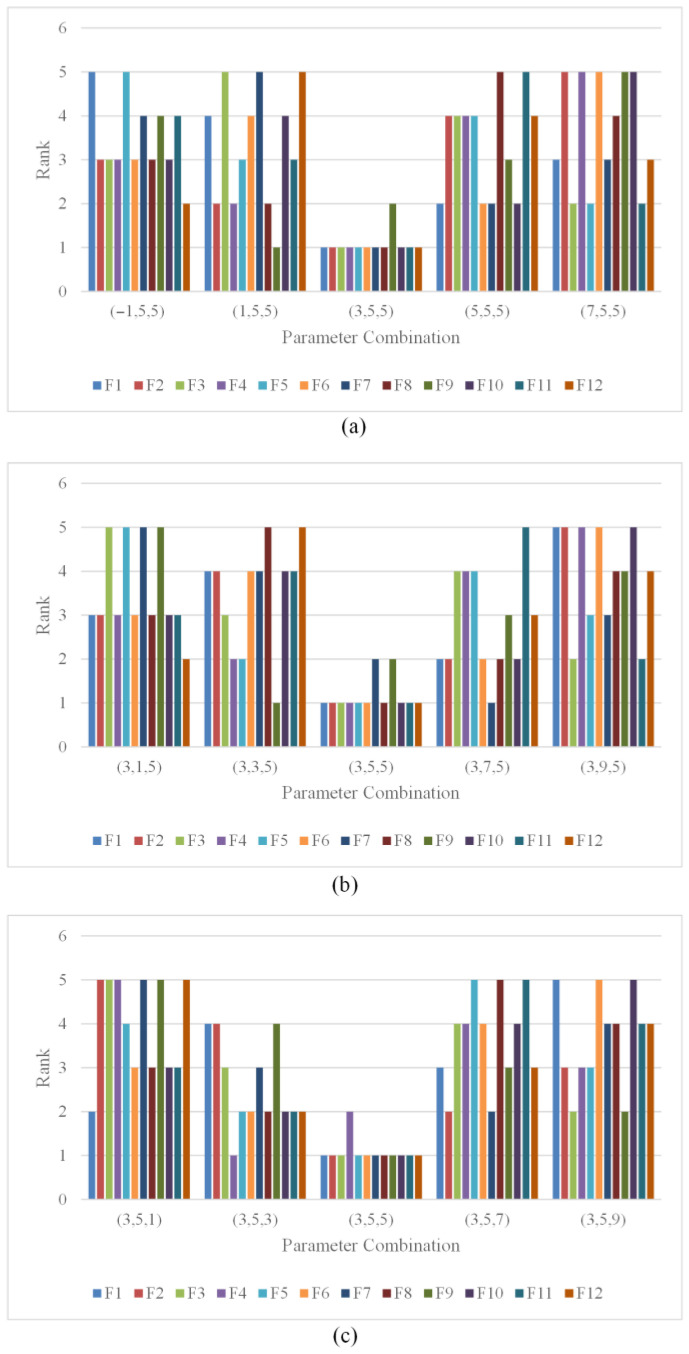
The decay factor corresponds to the ranking on different parameter combinations.

This section addresses the configuration of the decay factor’s pivotal parameters: λ, α, and β. We employ the control variable method to determine the specific values of these parameters. Firstly, to ascertain the value of parameter λ, we assume it is defined within the set {−1, 1, 3, 5, 7}. Meanwhile, we set the values of α and β to 5 each. This generates five parameter combinations: {−1, 5, 5}, {1, 5, 5}, {3, 5, 5}, {5, 5, 5} and {7, 5, 5}. Subsequently, we apply the NDFNGO algorithm corresponding to each of these five parameter combinations to solve 12 mural image segmentation problems. The algorithm’s fitness values across all twelve test problems were subsequently ranked, with comparative results visualized in Figure 8a. The data clearly indicates that configuration λ = 3 yields superior ranking performance. the NDFNGO algorithm achieves the highest fitness value ranking, demonstrating superior mural image segmentation performance. Therefore, parameter λ is determined to be 3 to ensure the algorithm’s image segmentation efficacy. Secondly, to determine the value of parameter α, we assume it is defined within the set {1, 3, 5, 7, 9}. Here, we set the values of λ and β to 3 and 5, respectively. This yields five parameter combinations: {3, 1, 5}, {3, 3, 5}, {3, 5, 5}, {3, 7, 5} and {3, 9, 5}. Subsequently, the NDFNGO algorithm was systematically applied with each parameter combination to the identical set of twelve mural segmentation tasks, followed by performance ranking based on obtained fitness values. The experimental results are shown in Figure 8b. The figure reveals that when parameter α is set to 5, the NDFNGO algorithm attains the highest fitness value ranking, indicating enhanced mural image segmentation performance. Consequently, parameter α is set to 5 to maintain the algorithm’s image segmentation capabilities. Finally, to ascertain the value of parameter β, we assume it is defined within the set {1, 3, 5, 7, 9}. In this case, we set the values of λ and α to 3 and 5, respectively. This results in five parameter combinations: {3, 5, 1}, {3, 5, 3}, {3, 5, 5}, {3, 5, 7} and {3, 5, 9}. Again, we apply the NDFNGO algorithm corresponding to each of these combinations to the 12 mural image segmentation problems and rank their fitness values. The experimental results are illustrated in Figure 8c. The figure indicates that when parameter β is set to 5, the NDFNGO algorithm achieves the highest fitness value ranking, showcasing improved mural image segmentation performance. Thus, parameter β is determined to be 5 to ensure the algorithm’s image segmentation performance. In summary, to guarantee the performance of the NDFNGO algorithm in solving mural image segmentation problems, we set the parameters as λ=3, α=5, and β=5.

### 5.3. Comparative Analysis of Linear Factors

**Figure 9:**
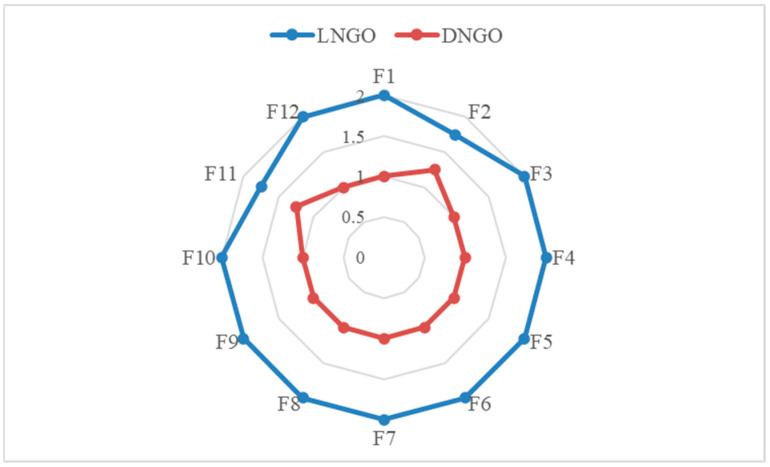
Ranking of linear and nonlinear factors in mural segmentation.

This section primarily analyzes the performance—enhancing effects of the proposed decay factor and the conventional linear decreasing factor on the NGO algorithm to verify the rationality of the decay factor introduced in this paper. Specifically, we define LNGO as the NGO algorithm augmented with the linear factor 1−t/T to control the global exploration and local exploitation phases. Similarly, we define DNGO as the NGO algorithm enhanced with the decay factor to regulate these two phases. Subsequently, we apply the resulting LNGO and DNGO algorithms to solve 12 mural image segmentation problems to evaluate the performance of the two control factors. The experimental results are presented in Figure 9. From the figure, it is visually evident that LNGO ranks lower than DNGO across all 12 mural image segmentation problems. This is primarily because, although the linear factor in LNGO has a simple structure, its linear distribution characteristic renders the algorithm almost incapable of local exploitation in the early iterations. This leads to a certain degree of accuracy loss during the initial stages of the algorithm. Meanwhile, in the later iterations, the algorithm’s global exploration capability is almost entirely compromised, preventing it from effectively escaping local optimal image segmentation threshold traps and thereby degrading the accuracy and quality of image segmentation. In contrast, the decay factor proposed in this paper fully takes into account the balance between the two phases during the algorithm’s iterative process. It enables the algorithm to possess a certain level of accuracy—capturing ability in the early iterations while also endowing it with the capacity to escape traps in the later iterations. As a result, the image segmentation effect is significantly improved.

### 5.4. Population Diversity Analysis

**Figure 10:**
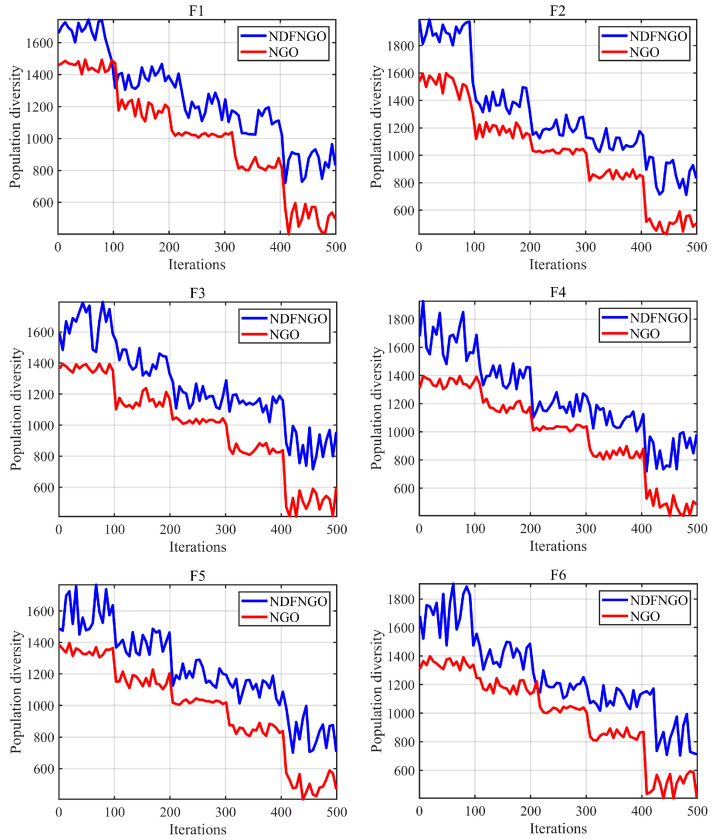
Algorithm population diversity.

This part centers on analyzing the population variety of the NDFNGO algorithm when addressing the mural image division task. Within the framework of optimization methods, great population variety indicates robust global exploration ability, enabling the algorithm to explore a broader solution scope. When used for mural image division, this trait helps efficiently recognize the best division thresholds, thus enhancing the overall division performance. The test outcomes are shown in Figure 10. The vertical axis stands for population variety, and the horizontal axis relates to the iteration count. The blue line shows how the population variety of the NDFNGO algorithm changes, while the red line demonstrates that of the initial NGO algorithm. A thorough breakdown of these outcomes is offered in the discussion that follows.

As displayed in the diagram, in the initial iteration phase, the NDFNGO algorithm sustains a comparatively elevated degree of population variety. This phenomenon mirrors the algorithm’s robust ability to probe the segmentation threshold domain right from the start and successfully steer clear of premature convergence. This advantage primarily stems from the nonlinear differential learning strategy proposed in this study, which significantly enhances the algorithm’s global exploration capability, enabling more comprehensive coverage of the solution space in mural image segmentation. As the iterations proceed, the population diversity of NDFNGO gradually decreases but at a slower rate compared with the original NGO algorithm, indicating a well-regulated balance between exploration and exploitation. This equilibrium allows the algorithm to sustain robust global search performance while gradually strengthening local refinement. The incorporation of the decay factor further contributes to this balance by harmonizing the transition between the two search phases, thereby reducing the risk of stagnation in locally optimal threshold regions. In the later stages of iteration, the population diversity of NDFNGO remains consistently higher and more stable than that of the original NGO, suggesting that the algorithm has successfully identified a promising region in the search space and continues to refine it through effective exploitation. Coupled with the fractional-order adaptive learning strategy, the algorithm achieves higher-quality segmentation thresholds for mural images. In summary, the integration of the three learning strategies in the proposed NDFNGO algorithm ensures sustained population diversity throughout the optimization process. Compared to the conventional NGO algorithm, NDFNGO demonstrates superior global exploration capability, enhanced ability to escape local optima, and improved overall performance in mural image segmentation tasks.

### 5.5. Exploration/Exploitation Balance Analysis

**Figure 11:**
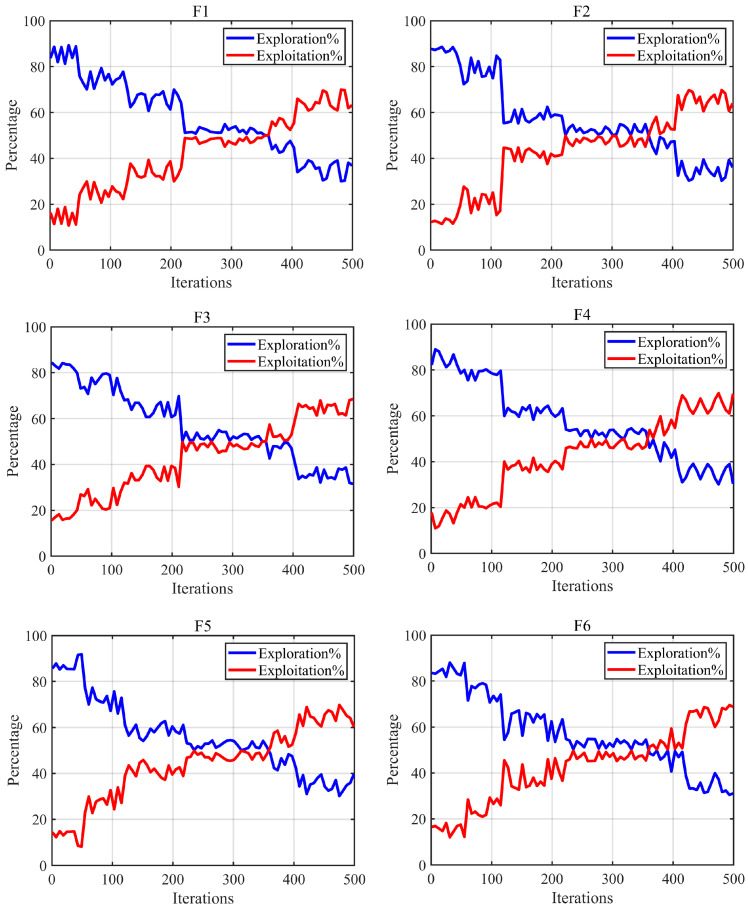
Algorithm exploration/exploitation ratio chart.

This part mainly centers on examining the equilibrium between the exploration and exploitation stages of the NDFNGO algorithm in tackling mural image segmentation tasks. A properly crafted optimization method ought to strike an appropriate balance between these two stages. In the initial iteration period, it should identify the optimal segmentation threshold area via global exploration; afterward, in the later iteration period, it should further polish the detected potential optimal segmentation threshold area through local exploitation to boost the quality of the segmentation thresholds. The test findings are shown in Figure 11. The horizontal axis stands for the number of iterations, and the vertical axis signifies the ratio of the two stages. The red line represents the local exploitation stage, while the blue line denotes the global exploration stage. A detailed breakdown of the balance between these stages is provided in the following content.

As can be seen from the figure, during the early iterations of the NDFNGO algorithm, the global exploration phase dominates, indicating that the algorithm can effectively locate potential optimal segmentation threshold regions in its initial stages, thereby increasing the likelihood of discovering the optimal segmentation thresholds. This is primarily attributed to the proposed nonlinear differential learning strategy, which, in combination with individual differences, effectively enhances the exploration step size during the search for segmentation thresholds, enabling the algorithm to identify superior solution regions. As iterations progress, the local exploitation capability of the NDFNGO algorithm gradually improves. By mid-iteration, a balance is achieved between the global exploration and local exploitation phases, during which both high global exploration and local exploitation rates are maintained. This harmonious coordination between the two phases in the solution search process enhances the quality of mural image segmentation, largely thanks to the proposed decay factor, which achieves this balance through adaptability. In the later stages of iteration, local exploitation takes precedence, allowing the algorithm to deeply explore the potential segmentation threshold regions identified earlier and refine the segmentation threshold accuracy, thereby effectively improving the performance of mural image segmentation. This is mainly due to the proposed Fractional-order adaptive learning strategy, which, combined with historical individual information, effectively enhances the algorithm’s local exploitation capability. However, it is noteworthy that, despite the dominance of the local exploitation phase in the later iterations, the NDFNGO algorithm still retains a certain degree of global exploration capability, significantly mitigating the risk of the algorithm being trapped in local optima. In summary, the three learning strategies proposed in this paper balance the algorithm’s global exploration and local exploitation capabilities from different perspectives, enabling the proposed NDFNGO algorithm to effectively solve the mural image segmentation problem.

### 5.6. Run Parameter Settings

**Figure 12:**
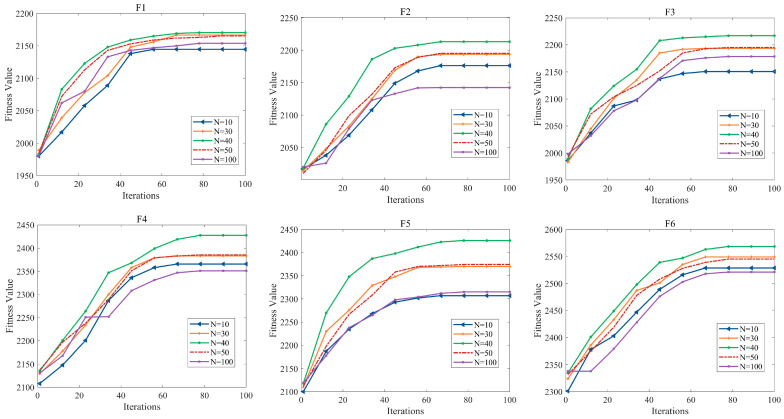
Convergence curves of algorithms under different population size settings.

This part mainly centers on examining the internal parameters of the NDFNGO algorithm in addressing mural image segmentation tasks, placing special focus on two core parameters: population scale and maximum iteration number. Here, we delve into the sensitivity and specific settings of these two parameters. Firstly, we set the population sizes to 10, 30, 40, 50, and 100, respectively. Subsequently, under the condition of a maximum iteration count of 100, we conduct mural image segmentation experiments. Each experiment is independently and non-repetitively executed 30 times, and the average fitness function value is calculated. The changes in the fitness function value with respect to the iteration count under different population size settings are depicted in Figure 12. From the figure, it can be observed that in the mural segmentation experiments conducted under various population size settings, the NDFNGO algorithm tends to exhibit a certain stable convergence trend in most cases when the iteration count reaches 70. As iterations progress, this stable trend becomes increasingly pronounced. From a statistical perspective, this stable trend is meaningful. Therefore, to conserve computational costs, we set the maximum iteration count to 100. The segmentation results obtained after 100 iterations are stable and meaningful. Simultaneously, the figure also reveals that under different population size settings, the fitness function values corresponding to population sizes of 10 and 100 are relatively poor. This is primarily because a smaller population size is not conducive to global exploration, leading the algorithm to easily fall into local optimal segmentation thresholds. By contrast, an expanded population scale cannot guarantee the concentration capability of solutions, which in turn impacts the outcomes of image segmentation. Moreover, a more in-depth investigation reveals that the algorithm’s performance remains inferior when the population scale is set to 30 or 50, compared to when it is set to 40. Thus, in the context of this research, setting the population scale to 40 better brings out the mural image segmentation performance of the NDFNGO algorithm. To sum up, for the subsequent experiments in this study, the population scale is set to 40 and the maximum iteration number is set to 100.

### 5.7. Ablation on Strategies Effectiveness

**Figure 13:**
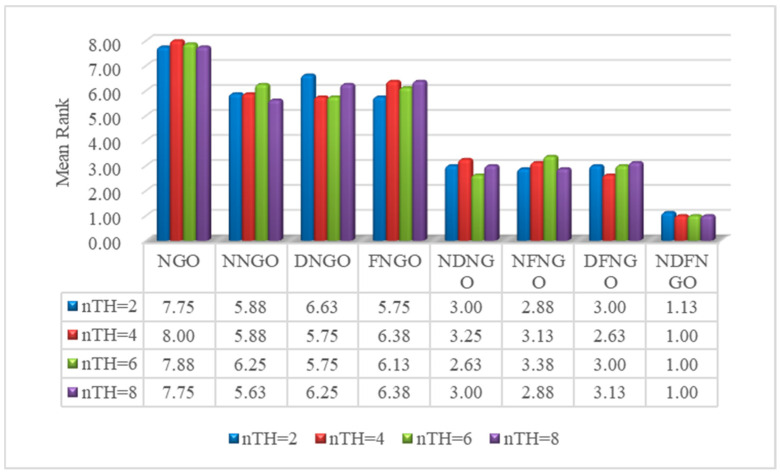
Ablation chart for strategy effectiveness.

This section primarily focuses on evaluating the performance—enhancing effects and synergistic interactions of the three improved strategies proposed in this paper, namely the nonlinear differential learning strategy, decay factor, and fractional—order adaptive learning strategy, on algorithm performance. To conduct strategy ablation experiments, we define the following: integrating the nonlinear differential learning strategy into the NGO algorithm forms NNGO; incorporating the decay factor into the NGO algorithm creates DNGO; and embedding the fractional—order adaptive learning strategy into the NGO algorithm results in FNGO. Furthermore, integrating the nonlinear differential learning strategy and the decay factor into the NGO gives rise to NDNGO; combining the nonlinear differential learning strategy and the fractional—order adaptive learning strategy into the NGO algorithm forms NFNGO; and integrating the decay factor and the fractional—order adaptive learning strategy into the NGO algorithm yields DFNGO. Additionally, the NDFNGO algorithm is formed by simultaneously introducing all three improved strategies. Subsequently, experiments were conducted on twelve mural image segmentation problems to assess the effectiveness and synergistic effects of these strategies. The experimental results are shown in Figure 13. As can be seen from the figure, the NNGO, DNGO, and FNGO algorithms all outperform the original NGO algorithm in terms of average ranking across different segmentation thresholds. This is mainly because the three improved strategies proposed in this study enhance the performance of the NGO algorithm from the perspectives of global exploration, algorithm balance, and local exploitation, respectively, leading to a certain improvement in its image segmentation performance. This phenomenon confirms the effectiveness of the three improved strategies proposed in this study. Secondly, the performance of the NDNGO, NFNGO, and DFNGO algorithms, which are formed by the synergistic combination of two improved strategies, is superior to that of the algorithms formed by introducing a single improved strategy. This indicates that the improved strategies proposed in this study are progressive, and they can work synergistically to mutually promote the enhancement of the algorithm’s global exploration phase, local exploitation phase, and balance performance, effectively improving the mural image segmentation performance. Finally, as shown in the figure, the NDFNGO algorithm achieves the best ranking in mural image segmentation. This further confirms the synergistic effect of the strategies proposed in this study, as the further synergy of the three improved strategies leads to a further improvement in the algorithm’s mural image segmentation performance. The above—mentioned experimental phenomena confirm the effectiveness of the improved strategies proposed in this paper and also demonstrate the existence of a performance—promoting synergistic effect among the three strategies.

### 5.8. Fitness Function Values Analysis

This part centers on examining the fitness function values of the NDFNGO algorithm when tackling mural image segmentation tasks, with the aim of evaluating its overall segmentation capability. The test results are organized in Table 3. In this table, Mean refers to the average fitness function value gained from 30 independent trials, Std stands for the standard deviation of these values, Mean Rank denotes the algorithm’s average position across twelve mural image segmentation assignments, and Final Rank represents its overall position based on the Mean Rank measure. In addition, a Wilcoxon rank-sum test was performed on the fitness function values derived from the 30 independent experiments to statistically validate the results. To minimize the risk of false-positive conclusions, multiple hypothesis testing corrections were applied. The corresponding statistical results are provided in Table 4.

**Figure 14:**
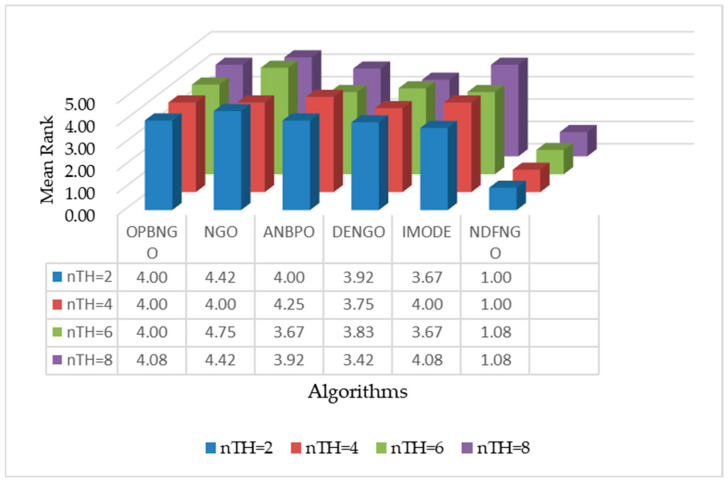
Ranking of fitness function values.

As can be seen from Table 3, when the number of segmentation thresholds is 2, the NDFNGO algorithm ranks first among all twelve mural image segmentation problems, achieving a 100%-win rate compared to competing algorithms. This is mainly because three learning strategies have been incorporated, which have boosted the algorithm’s searching capabilities and allowed for effective exploration within the combination of segmentation thresholds. When the count of segmentation thresholds is raised to 4, the NDFNGO algorithm still takes first place in all twelve tasks, showing better performance in comparison to other competing algorithms. When the segmentation threshold is set to 6, the NDFNGO algorithm takes first place in 11 out of 12 mural image segmentation tasks, boasting a win rate of 91.7%. It only performs marginally worse than ONBNGO on the F4 task. This shows that while the NDFNGO algorithm delivers excellent image segmentation results, it still has room for enhancement in some specific tasks. When the number of segmentation thresholds reaches 8, the NDFNGO algorithm secures first place in 11 tasks, keeping a 91.7% win rate and generally outperforming other competing algorithms—though it fails to outdo DENGO on the F1 task. This occurrence implies that the NDFNGO algorithm still has certain constraints in specific scenarios. From a holistic view, however, across four different numbers of segmentation thresholds, the NDFNGO algorithm achieves a 95.85% win rate against competing algorithms, verifying its outstanding performance in mural image segmentation. Additionally, to visually showcase the NDFNGO algorithm’s edge in fitness function values, Figure 14 presents the algorithm’s average ranking under different segmentation thresholds. As shown in the figure, under the four threshold conditions, the NDFNGO algorithm’s average rankings are 1.00, 1.00, 1.08, and 1.08, respectively, proving it has stronger mural image segmentation capabilities than competing algorithms. Furthermore, in the conducted Wilcoxon rank-sum tests, the NDFNGO algorithm outperforms OPBNGO and DENGO significantly in 47 out of 48 experiments, hitting a 97.9% win rate. It also outperforms NGO, ANBPO, and IMODE significantly in all 48 experiments, with a win rate over 95%. In conclusion, the NDFNGO algorithm proposed in this paper performs exceptionally well in mural image segmentation tasks and can be regarded as a promising approach for mural image segmentation.

### 5.9. PSNR Metrics Analysis

This part provides an analysis of the Peak Signal-to-Noise Ratio (PSNR) values of the NDFNGO algorithm when applied to mural image segmentation, aiming to assess the level of image distortion generated during the segmentation process. The test findings are compiled in Table 5. Here, Mean refers to the average PSNR value gained from 30 independent trials, Std stands for the standard deviation of these PSNR values, Mean Rank represents the algorithm’s average position across twelve mural image segmentation assignments, and Final Rank reflects the overall position obtained from the Mean Rank measure. Additionally, a Wilcoxon rank-sum test was performed on the PSNR values collected from the 30 independent experiments to statistically assess the differences. To ensure the reliability of the results, multiple hypothesis testing corrections were applied to minimize potential false-positive conclusions. The corresponding statistical outcomes are presented in Table 6.

**Figure 15:**
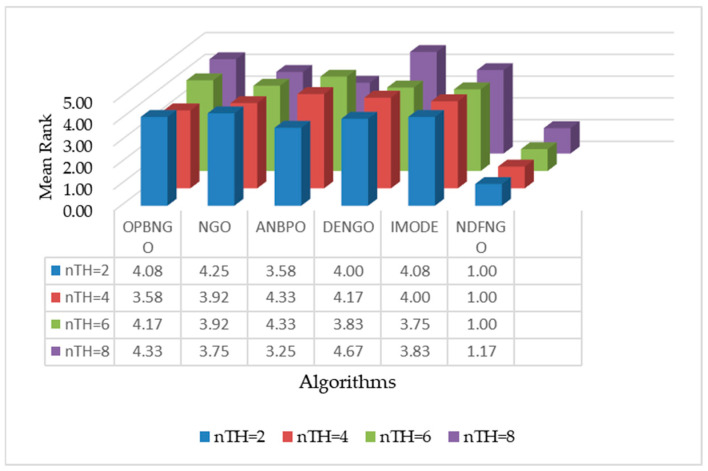
PSNR metrics rank.

As displayed in Table 5, when the count of segmentation thresholds is set to 2, the NDFNGO algorithm takes first place across all twelve mural image segmentation tasks, attaining a 100% win rate against competing algorithms. This exceptional performance is mainly due to the incorporation of the three proposed learning strategies—these strategies greatly boost the algorithm’s search efficiency and allow the segmented thresholds to preserve pixel information from the original image as much as possible. When the number of segmentation thresholds rises to 4, the NDFNGO algorithm keeps holding the top position in all twelve test scenarios, showing a lower image classification error rate than other comparable algorithms. Similarly, with 6 thresholds, it again secures first place in every problem, sustaining a 100% win rate and demonstrating a strong capacity to preserve the structural and pixel integrity of the original images—effectively reducing image distortion after segmentation. At 8 thresholds, the NDFNGO algorithm ranks first in eleven out of twelve problems, achieving a 91.6% win rate. Although its performance on the F1 problem is slightly inferior to that of the IMODE algorithm, it still surpasses other competitors in most cases. This minor performance gap suggests that while the NDFNGO algorithm is generally robust, certain image characteristics may still pose challenges in preserving pixel-level information. Overall, across the four threshold settings, NDFNGO attains an impressive 97.9% average win rate compared with other algorithms, confirming its superior ability to minimize image distortion and maintain original pixel features in mural image segmentation. To further illustrate this advantage, Figure 15 presents the average ranking of the NDFNGO algorithm under varying threshold numbers. The recorded average rankings—1.00, 1.00, 1.00, and 1.17—demonstrate its consistently strong performance in minimizing post-segmentation image distortion relative to competing methods. Moreover, results from the Wilcoxon rank-sum tests conducted on PSNR values reinforce these findings: NDFNGO significantly outperforms ANBPO and IMODE in 47 out of 48 experiments (a 97.9% win rate), and consistently surpasses OPBNGO, NGO, and DENGO in all 48 experiments, with a win rate exceeding 98%. In summary, the proposed NDFNGO algorithm exhibits exceptional performance in mural image segmentation tasks, achieving low image distortion, high pixel fidelity, and superior stability across multiple segmentation thresholds. These results confirm its potential as a highly effective and reliable method for mural image segmentation.

### 5.10. SSIM Metrics Analysis

This part examines the Structural Similarity Index (SSIM) values of the NDFNGO algorithm when addressing mural image segmentation tasks, with the goal of evaluating how effectively the algorithm preserves image structural information during segmentation. The test results are compiled in Table 7. Here, Mean stands for the average SSIM value derived from 30 independent tests, Std refers to the standard deviation of these SSIM values, Mean Rank denotes the algorithm’s average position across twelve mural image segmentation assignments, and Final Rank represents its overall position based on the Mean Rank measure. Additionally, a Wilcoxon rank-sum test was conducted on the SSIM values derived from the 30 independent experiments to statistically validate the results. To minimize the likelihood of false-positive conclusions, multiple hypothesis testing corrections were applied. The corresponding statistical results are provided in Table 8.

**Figure 16:**
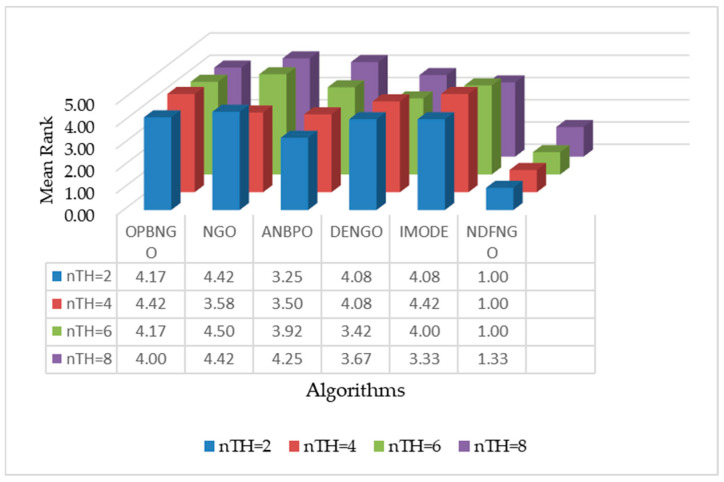
SSIM metrics rank.

As displayed in Table 7, when the count of segmentation thresholds is set to 2, the NDFNGO algorithm takes first place across all twelve mural image segmentation assignments, attaining a 100% win rate against competing algorithms. This excellent performance is mainly due to the incorporation of the three proposed learning strategies—these strategies greatly boost the algorithm’s search efficiency and allow the segmentation thresholds to preserve the original image’s structural and spatial layout information as much as possible. When the number of segmentation thresholds rises to 4, the NDFNGO algorithm steadily keeps its top position across all twelve tasks. Compared with other algorithms, it exhibits a lower rate of structural information loss and provides better preservation of image integrity in the segmented outputs. Similarly, when the number of thresholds is 6, NDFNGO continues to rank first in all twelve problems, again achieving a 100% win rate, thereby demonstrating its strong ability to maintain structural consistency and segmentation accuracy. At 8 thresholds, the NDFNGO algorithm secures first place in eleven out of twelve problems, corresponding to a 91.6% win rate. Although its performance on the F1 problem is slightly inferior to that of OPBNGO and ANBPO, it still surpasses all other algorithms in most cases. This minor performance drop indicates that while NDFNGO performs robustly overall, it may encounter challenges in preserving structural information for specific image types. From an overall perspective, across all four segmentation threshold configurations, the NDFNGO algorithm achieves an impressive 97.9% average win rate compared with competing algorithms. These results confirm its outstanding ability to preserve structural features and minimize the loss of image integrity in mural image segmentation. To visually illustrate this advantage, Figure 16 presents the average SSIM ranking of the NDFNGO algorithm under different segmentation thresholds. The corresponding rankings—1.00, 1.00, 1.00, and 1.33—further demonstrate its superior capability in maintaining the structural fidelity of the original image compared with other methods. From an overall perspective, across all four segmentation threshold configurations, the NDFNGO algorithm achieves an impressive 97.9% average win rate compared with competing algorithms. These results confirm its outstanding ability to preserve structural features and minimize the loss of image integrity in mural image segmentation. To visually illustrate this advantage, Figure 16 presents the average SSIM ranking of the NDFNGO algorithm under different segmentation thresholds. The corresponding rankings—1.00, 1.00, 1.00, and 1.33—further demonstrate its superior capability in maintaining the structural fidelity of the original image compared with other methods. Moreover, results from the Wilcoxon rank-sum tests conducted on SSIM values reinforce these findings: the NDFNGO algorithm significantly outperforms OPBNGO, ANBPO, DENGO, and IMODE in 47 out of 48 experiments, achieving a 97.9% win rate, and exceeds NGO in all 48 experiments, with a win rate above 95%. In conclusion, the proposed NDFNGO algorithm exhibits exceptional structural preservation capability and segmentation performance across various threshold levels. Its ability to maintain high structural similarity and low distortion makes it a promising and effective approach for mural image segmentation.

### 5.11. FSIM Metrics Analysis

This part examines the Feature Similarity Index (FSIM) values of the NDFNGO algorithm when addressing mural image segmentation tasks, aiming to evaluate the algorithm’s capability to retain key image feature information during the segmentation process. The test findings are compiled in Table 9. Here, Mean refers to the average FSIM value gained from 30 independent trials, Std stands for the standard deviation of these FSIM values, Mean Rank represents the algorithm’s average position across twelve mural image segmentation assignments, and Final Rank reflects its overall position obtained from the Mean Rank measure. Additionally, a Wilcoxon rank-sum test was performed on the FSIM values obtained from the 30 independent experiments to statistically validate the outcomes. To ensure robustness and avoid false-positive results, multiple hypothesis testing corrections were applied. The corresponding statistical findings are reported in Table 10.

**Figure 17:**
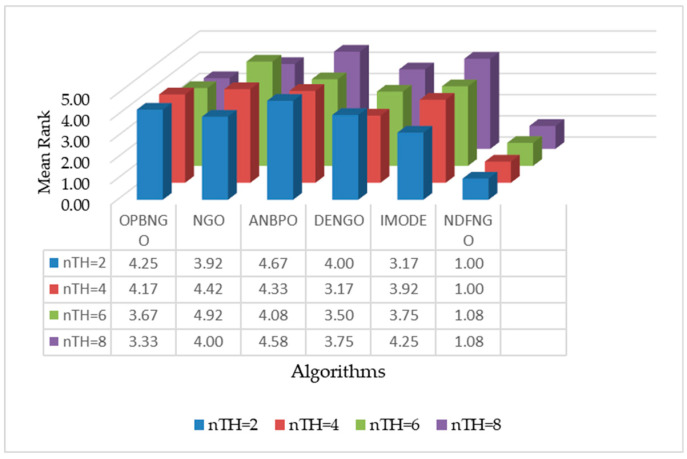
FSIM metrics rank.

As displayed in Table 9, when the count of segmentation thresholds is set to 2, the NDFNGO algorithm claims the top spot across all twelve mural image segmentation tasks, attaining a 100% win rate against competing algorithms. This excellent performance is mainly due to the incorporation of the three proposed learning strategies—these strategies greatly boost the algorithm’s search ability and allow the segmentation thresholds to maximize the preservation of local feature information from the original images. When the number of segmentation thresholds rises to 4, the NDFNGO algorithm steadily holds first place in all twelve test scenarios. Compared with competing algorithms, it achieves a lower feature distortion rate and demonstrates stronger preservation of local feature characteristics in the segmented results. When the threshold number reaches 6, the NDFNGO algorithm ranks first in eleven out of twelve problems, attaining a 91.6% win rate. This result highlights its strong capacity for maintaining local feature similarity and ensuring a high degree of correspondence between the segmented and original images. When the number of segmentation thresholds increases to 4, the NDFNGO algorithm consistently maintains first place in all twelve test cases. Compared with competing algorithms, it achieves a lower feature distortion rate and demonstrates stronger preservation of local feature characteristics in the segmented results. When the threshold number reaches 6, the NDFNGO algorithm ranks first in eleven out of twelve problems, attaining a 91.6% win rate. This result highlights its strong capacity for maintaining local feature similarity and ensuring a high degree of correspondence between the segmented and original images. At 8 thresholds, the algorithm again ranks first in eleven out of twelve cases, also with a 91.6% win rate. Although its performance on the F1 problem is slightly lower than that of OPBNGO, it continues to outperform the remaining algorithms across most test cases. This minor deviation suggests that while the NDFNGO algorithm is generally robust, certain image characteristics may still present challenges for local feature preservation. Overall, considering all four segmentation threshold settings, the NDFNGO algorithm achieves an impressive 95.8% average win rate compared with competing methods. These findings confirm its remarkable ability to retain local feature information and minimize feature loss during mural image segmentation. To further illustrate this advantage, Figure 17 presents the algorithm’s average FSIM rankings under varying threshold conditions. The recorded averages—1.00, 1.00, 1.08, and 1.08—demonstrate its consistently strong performance in reducing local feature loss compared with other algorithms. Moreover, results from the Wilcoxon rank-sum tests conducted on FSIM values further support these conclusions: NDFNGO significantly outperforms NGO and ANBPO in 47 out of 48 experiments, achieving a 97.9% win rate, and surpasses OPBNGO, DENGO, and IMODE in all 48 experiments, with a win rate exceeding 95%. In summary, the proposed NDFNGO algorithm exhibits outstanding feature preservation and segmentation accuracy across various threshold levels. Its ability to maintain local image detail and minimize feature loss makes it a highly effective and promising method for mural image segmentation.

### 5.12. Comprehensive Metric Analysis

**Figure 18:**
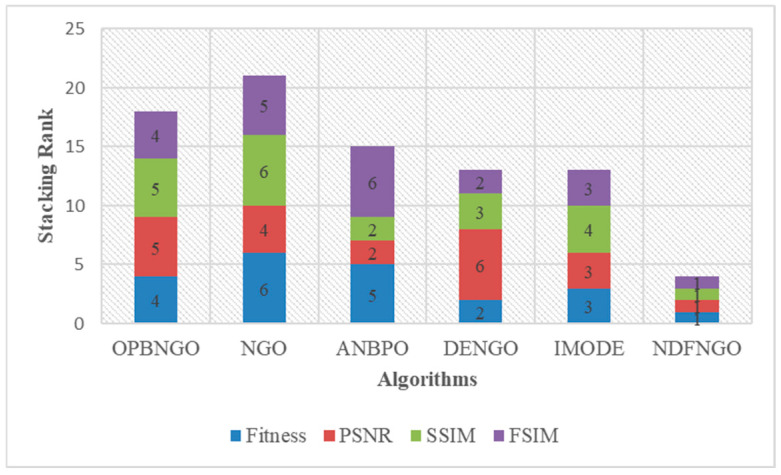
Algorithm stacking ranking chart.

The previous parts have separately analyzed the fitness function values, PSNR values, SSIM values, and FSIM values of the NDFNGO algorithm when tackling mural image segmentation tasks. In this part, the main focus is on conducting a comprehensive analysis of these four key metrics to assess the mural segmentation performance of the NDFNGO algorithm. The experimental outcomes are shown in Figure 18. As observed from the figure, the NDFNGO algorithm has lower bar heights in comparison to competing algorithms. Specifically, it outperforms the IMODE algorithm— which ranks second— by 69.23%, proving its superior overall performance in mural image segmentation. This result is mainly due to the notable improvements in the algorithm’s segmentation threshold search capability, which are driven by the nonlinear differential learning strategy, decay factor, and fractional-order adaptive learning strategy proposed in this paper. These enhancements enable more rational selection of segmentation thresholds, thereby boosting the algorithm’s performance in addressing mural image segmentation tasks and achieving higher-quality image segmentation. Consequently, the NDFNGO algorithm can be regarded as a promising option for mural image segmentation.

## 6. Conclusions and Future Works

When applied to mural image segmentation, the original NGO algorithm has shortcomings in global exploration, local exploitation, and maintaining a proper balance between these two processes—often leading to less-than-ideal segmentation similarity. To resolve these issues, this paper proposes an enhanced algorithm called NDFNGO. It integrates three improvement strategies: a nonlinear differential learning strategy, a decay factor, and a fractional-order adaptive learning strategy, all developed to boost image segmentation performance. First, to address the original NGO algorithm’s inadequate global exploration capability, a nonlinear differential learning strategy is incorporated to strengthen its global search ability. By utilizing the adaptability and diversity of the differential mechanism, this strategy effectively enhances the algorithm’s capacity to find optimal segmentation threshold regions. Second, to alleviate the imbalance between global exploration and local exploitation, a decay factor is introduced. Through the nonlinear influence of exponential, cosine, and arctangent functions, this mechanism achieves a more harmonious transition between the two phases, enhancing search stability. Finally, to compensate for the limited local exploitation capacity, a fractional-order adaptive learning strategy is implemented, utilizing historical individual information to refine the local search process and further improve segmentation threshold quality. Extensive experiments conducted on twelve mural image segmentation problems validate the effectiveness of the proposed method. The NDFNGO algorithm demonstrates superior global search performance and achieves win rates of 95.85%, 97.9%, 97.9%, and 95.8% for the fitness function, PSNR, SSIM, and FSIM metrics, respectively. These results confirm its remarkable capability in preserving image structure and feature information, highlighting NDFNGO as a powerful and promising approach for mural image segmentation tasks.

Although the NDFNGO algorithm proposed in this paper has achieved promising optimization results, there are still certain limitations in its performance. Therefore, our subsequent work will focus on the following three aspects: (1) As NDFNGO is a combinatorial optimization algorithm, future efforts should be directed towards applying it to a broader range of combinatorial optimization problems to expand its application domains. (2) We will construct more mural image datasets and conduct performance analyses using NDFNGO to facilitate advancements in image restoration. (3) We aim to develop a multi-objective version of NDFNGO to make it suitable for solving a more diverse set of combinatorial optimization problems.

## Figures and Tables

**Table 1:** Objective function values for engineering design optimization problems.

Problems	Metrics	OPBNGO	NGO	ANBPO	DENGO	IMODE	NDFNGO
TCSDcase	Mean	0.0131	0.0129	0.0127	0.0157	0.0142	0.0127
	Std	9.4069 × 10^−04^	3.4657 × 10^−04^	8.3209 × 10^−06^	2.1191 × 10^−03^	1.3389 × 10^−03^	4.9656 × 10^−08^
WBD	Mean	1.6821	1.6702	1.6702	1.9266	1.8660	1.6702
	Std	3.7290 × 10^−02^	2.4247 × 10^−05^	1.8647 × 10^−08^	9.6425 × 10^−02^	9.6793 × 10^−02^	1.9340 × 10^−16^
TenBTD	Mean	528.8000	527.3900	524.7400	558.5300	738.2100	524.4800
	Std	2.9888 × 10^+00^	2.9417 × 10^+00^	3.9746 × 10^−01^	8.3592 × 10^+00^	6.8925 × 10^+01^	4.0911 × 10^−02^
TBTDP	Mean	263.9000	263.9000	263.9000	266.4200	264.4100	263.9000
	Std	2.5972 × 10^−04^	4.7756 × 10^−04^	3.1531 × 10^−12^	2.9474 × 10^+00^	6.7010 × 10^−01^	0.0000 × 10^+00^

**Table 3:** Fitness function value.

Image	nTH	OPBNGO		NGO		ANBPO		DENGO		IMODE		NDFNGO	
		Mean	Std	Mean	Std	Mean	Std	Mean	Std	Mean	Std	Mean	Std
F1	2	1.868 × 10^+03^	1.436 × 10^−01^	1.812 × 10^+03^	9.825 × 10^−01^	1.750 × 10^+03^	7.935 × 10^−01^	1.741 × 10^+03^	2.075 × 10^−01^	1.857 × 10^+03^	1.280 × 10^−01^	1.921 × 10^+03^	6.792 × 10^−03^
	4	1.970 × 10^+03^	8.097 × 10^−01^	1.996 × 10^+03^	7.163 × 10^−01^	1.918 × 10^+03^	1.802 × 10^−01^	1.903 × 10^+03^	5.640 × 10^−01^	1.969 × 10^+03^	5.685 × 10^−01^	2.084 × 10^+03^	2.144 × 10^−03^
	6	2.027 × 10^+03^	5.658 × 10^−01^	2.028 × 10^+03^	9.724 × 10^−01^	2.047 × 10^+03^	3.306 × 10^−02^	2.052 × 10^+03^	1.694 × 10^−01^	2.066 × 10^+03^	1.668 × 10^−01^	2.197 × 10^+03^	4.662 × 10^−03^
	8	2.125 × 10^+03^	5.934 × 10^−02^	2.147 × 10^+03^	1.576 × 10^−01^	2.107 × 10^+03^	5.240 × 10^−01^	2.174 × 10^+03^	9.556 × 10^−01^	2.169 × 10^+03^	3.412 × 10^−02^	2.170 × 10^+03^	1.220 × 10^−03^
F2	2	1.862 × 10^+03^	5.750 × 10^−01^	1.876 × 10^+03^	8.802 × 10^−01^	1.862 × 10^+03^	7.863 × 10^−01^	1.714 × 10^+03^	2.514 × 10^−02^	1.756 × 10^+03^	6.524 × 10^−01^	1.943 × 10^+03^	8.818 × 10^−03^
	4	1.995 × 10^+03^	8.161 × 10^−01^	1.972 × 10^+03^	9.189 × 10^−01^	1.987 × 10^+03^	2.256 × 10^−01^	1.982 × 10^+03^	5.538 × 10^−01^	1.991 × 10^+03^	9.564 × 10^−01^	2.024 × 10^+03^	7.479 × 10^−03^
	6	2.009 × 10^+03^	3.430 × 10^−01^	2.004 × 10^+03^	1.678 × 10^−01^	2.049 × 10^+03^	7.734 × 10^−01^	2.038 × 10^+03^	3.866 × 10^−01^	2.016 × 10^+03^	4.290 × 10^−01^	2.148 × 10^+03^	6.244 × 10^−03^
	8	2.186 × 10^+03^	6.211 × 10^−01^	2.178 × 10^+03^	9.082 × 10^−02^	2.195 × 10^+03^	3.377 × 10^−01^	2.155 × 10^+03^	4.249 × 10^−01^	2.192 × 10^+03^	9.347 × 10^−01^	2.213 × 10^+03^	9.620 × 10^−03^
F3	2	1.851 × 10^+03^	6.881 × 10^−01^	1.849 × 10^+03^	8.050 × 10^−01^	1.829 × 10^+03^	5.729 × 10^−01^	1.836 × 10^+03^	6.558 × 10^−01^	1.898 × 10^+03^	5.898 × 10^−01^	1.961 × 10^+03^	5.569 × 10^−03^
	4	1.949 × 10^+03^	4.491 × 10^−01^	1.984 × 10^+03^	9.164 × 10^−01^	1.988 × 10^+03^	1.916 × 10^−01^	1.955 × 10^+03^	3.579 × 10^−01^	1.938 × 10^+03^	1.699 × 10^−01^	2.058 × 10^+03^	2.752 × 10^−02^
	6	2.030 × 10^+03^	8.881 × 10^−01^	2.012 × 10^+03^	6.892 × 10^−01^	2.024 × 10^+03^	9.621 × 10^−01^	2.074 × 10^+03^	4.924 × 10^−01^	2.047 × 10^+03^	8.865 × 10^−01^	2.180 × 10^+03^	1.355 × 10^−01^
	8	2.151 × 10^+03^	7.879 × 10^−01^	2.135 × 10^+03^	5.473 × 10^−01^	2.152 × 10^+03^	2.532 × 10^−01^	2.175 × 10^+03^	1.880 × 10^−01^	2.108 × 10^+03^	6.841 × 10^−01^	2.217 × 10^+03^	1.978 × 10^−01^
F4	2	1.949 × 10^+03^	5.573 × 10^−01^	1.951 × 10^+03^	8.416 × 10^−01^	2.092 × 10^+03^	8.822 × 10^−01^	2.011 × 10^+03^	4.750 × 10^−01^	1.975 × 10^+03^	8.280 × 10^−01^	2.163 × 10^+03^	9.956 × 10^−03^
	4	2.130 × 10^+03^	1.195 × 10^−01^	2.113 × 10^+03^	2.342 × 10^−01^	2.144 × 10^+03^	3.572 × 10^−02^	2.169 × 10^+03^	3.553 × 10^−01^	2.133 × 10^+03^	1.365 × 10^−02^	2.235 × 10^+03^	4.224 × 10^−03^
	6	2.294 × 10^+03^	7.226 × 10^−01^	2.224 × 10^+03^	3.939 × 10^−01^	2.240 × 10^+03^	7.516 × 10^−02^	2.263 × 10^+03^	5.199 × 10^−01^	2.269 × 10^+03^	4.632 × 10^−01^	2.290 × 10^+03^	1.482 × 10^−03^
	8	2.396 × 10^+03^	5.077 × 10^−01^	2.383 × 10^+03^	4.632 × 10^−01^	2.324 × 10^+03^	6.963 × 10^−01^	2.385 × 10^+03^	1.656 × 10^−01^	2.351 × 10^+03^	7.580 × 10^−01^	2.428 × 10^+03^	5.469 × 10^−04^
F5	2	1.939 × 10^+03^	8.780 × 10^−01^	1.932 × 10^+03^	9.665 × 10^−01^	1.943 × 10^+03^	7.130 × 10^−01^	2.046 × 10^+03^	2.864 × 10^−02^	2.072 × 10^+03^	9.764 × 10^−01^	2.178 × 10^+03^	3.565 × 10^−04^
	4	2.104 × 10^+03^	6.893 × 10^−03^	2.158 × 10^+03^	6.308 × 10^−01^	2.191 × 10^+03^	3.083 × 10^−01^	2.181 × 10^+03^	8.016 × 10^−01^	2.147 × 10^+03^	3.722 × 10^−01^	2.209 × 10^+03^	7.847 × 10^−03^
	6	2.273 × 10^+03^	3.353 × 10^−01^	2.233 × 10^+03^	5.279 × 10^−01^	2.269 × 10^+03^	4.237 × 10^−01^	2.235 × 10^+03^	9.597 × 10^−01^	2.229 × 10^+03^	7.703 × 10^−01^	2.345 × 10^+03^	9.597 × 10^−03^
	8	2.307 × 10^+03^	6.411 × 10^−01^	2.370 × 10^+03^	2.398 × 10^−01^	2.354 × 10^+03^	4.410 × 10^−01^	2.304 × 10^+03^	9.145 × 10^−01^	2.315 × 10^+03^	2.322 × 10^−01^	2.426 × 10^+03^	9.472 × 10^−03^
F6	2	2.161 × 10^+03^	3.758 × 10^−01^	2.204 × 10^+03^	2.140 × 10^−01^	2.101 × 10^+03^	7.289 × 10^−01^	2.261 × 10^+03^	3.895 × 10^−01^	2.272 × 10^+03^	2.227 × 10^−01^	2.380 × 10^+03^	5.454 × 10^−03^
	4	2.315 × 10^+03^	2.029 × 10^−01^	2.307 × 10^+03^	3.005 × 10^−02^	2.371 × 10^+03^	5.266 × 10^−01^	2.315 × 10^+03^	6.845 × 10^−01^	2.389 × 10^+03^	7.657 × 10^−01^	2.445 × 10^+03^	1.819 × 10^−03^
	6	2.418 × 10^+03^	9.388 × 10^−01^	2.483 × 10^+03^	8.073 × 10^−01^	2.469 × 10^+03^	4.836 × 10^−01^	2.472 × 10^+03^	4.621 × 10^−01^	2.474 × 10^+03^	9.264 × 10^−01^	2.513 × 10^+03^	6.420 × 10^−03^
	8	2.529 × 10^+03^	6.009 × 10^−02^	2.509 × 10^+03^	6.941 × 10^−01^	2.511 × 10^+03^	3.436 × 10^−02^	2.545 × 10^+03^	5.293 × 10^−01^	2.521 × 10^+03^	2.472 × 10^−01^	2.568 × 10^+03^	1.745 × 10^−03^
F7	2	1.877 × 10^+03^	7.012 × 10^−01^	1.851 × 10^+03^	2.791 × 10^−01^	1.804 × 10^+03^	7.594 × 10^−01^	1.748 × 10^+03^	5.869 × 10^−01^	1.768 × 10^+03^	7.637 × 10^−01^	1.992 × 10^+03^	9.866 × 10^−03^
	4	1.987 × 10^+03^	8.641 × 10^−01^	1.996 × 10^+03^	3.896 × 10^−01^	1.908 × 10^+03^	5.693 × 10^−01^	1.961 × 10^+03^	1.467 × 10^−01^	1.973 × 10^+03^	1.008 × 10^−01^	2.045 × 10^+03^	4.586 × 10^−03^
	6	2.091 × 10^+03^	5.088 × 10^−01^	2.023 × 10^+03^	6.092 × 10^−01^	2.037 × 10^+03^	5.751 × 10^−01^	2.059 × 10^+03^	5.536 × 10^−02^	2.048 × 10^+03^	5.382 × 10^−01^	2.143 × 10^+03^	2.119 × 10^−03^
	8	2.102 × 10^+03^	9.334 × 10^−01^	2.173 × 10^+03^	2.125 × 10^−01^	2.113 × 10^+03^	9.127 × 10^−01^	2.135 × 10^+03^	6.418 × 10^−01^	2.162 × 10^+03^	1.259 × 10^−01^	2.248 × 10^+03^	4.798 × 10^−01^
F8	2	2.107 × 10^+03^	6.872 × 10^−01^	2.205 × 10^+03^	2.521 × 10^−01^	2.252 × 10^+03^	7.299 × 10^−01^	2.221 × 10^+03^	3.558 × 10^−01^	2.142 × 10^+03^	2.216 × 10^−01^	2.336 × 10^+03^	7.602 × 10^−01^
	4	2.392 × 10^+03^	8.887 × 10^−01^	2.349 × 10^+03^	1.340 × 10^−01^	2.311 × 10^+03^	7.907 × 10^−02^	2.328 × 10^+03^	5.869 × 10^−01^	2.375 × 10^+03^	5.027 × 10^−01^	2.405 × 10^+03^	5.920 × 10^−03^
	6	2.401 × 10^+03^	2.753 × 10^−01^	2.410 × 10^+03^	2.295 × 10^−01^	2.465 × 10^+03^	4.047 × 10^−01^	2.427 × 10^+03^	6.835 × 10^−02^	2.439 × 10^+03^	6.842 × 10^−01^	2.535 × 10^+03^	2.325 × 10^−03^
	8	2.540 × 10^+03^	5.411 × 10^−01^	2.520 × 10^+03^	1.969 × 10^−01^	2.521 × 10^+03^	7.684 × 10^−01^	2.536 × 10^+03^	1.122 × 10^−01^	2.542 × 10^+03^	9.398 × 10^−02^	2.583 × 10^+03^	9.629 × 10^−04^
F9	2	1.798 × 10^+03^	8.340 × 10^−01^	1.716 × 10^+03^	4.649 × 10^−01^	1.890 × 10^+03^	9.573 × 10^−01^	1.874 × 10^+03^	8.512 × 10^−01^	1.798 × 10^+03^	4.318 × 10^−01^	1.970 × 10^+03^	7.397 × 10^−03^
	4	1.988 × 10^+03^	2.022 × 10^−01^	1.979 × 10^+03^	3.818 × 10^−01^	1.904 × 10^+03^	2.686 × 10^−01^	1.991 × 10^+03^	9.710 × 10^−01^	1.930 × 10^+03^	9.093 × 10^−01^	2.082 × 10^+03^	2.088 × 10^−03^
	6	2.092 × 10^+03^	2.431 × 10^−01^	2.084 × 10^+03^	8.573 × 10^−01^	2.074 × 10^+03^	9.516 × 10^−01^	2.063 × 10^+03^	8.121 × 10^−01^	2.017 × 10^+03^	6.737 × 10^−01^	2.110 × 10^+03^	5.475 × 10^−03^
	8	2.120 × 10^+03^	4.479 × 10^−02^	2.124 × 10^+03^	8.606 × 10^−02^	2.193 × 10^+03^	1.661 × 10^−01^	2.190 × 10^+03^	3.003 × 10^−01^	2.117 × 10^+03^	2.418 × 10^−01^	2.263 × 10^+03^	6.940 × 10^−03^
F10	2	1.787 × 10^+03^	1.176 × 10^−01^	1.745 × 10^+03^	7.112 × 10^−01^	1.800 × 10^+03^	9.043 × 10^−01^	1.880 × 10^+03^	6.732 × 10^−01^	1.873 × 10^+03^	7.050 × 10^−01^	1.979 × 10^+03^	2.886 × 10^−03^
	4	1.965 × 10^+03^	8.431 × 10^−01^	1.970 × 10^+03^	5.963 × 10^−01^	1.991 × 10^+03^	3.374 × 10^−01^	1.993 × 10^+03^	8.791 × 10^−01^	1.970 × 10^+03^	7.548 × 10^−01^	2.074 × 10^+03^	4.712 × 10^−04^
	6	2.043 × 10^+03^	9.635 × 10^−01^	2.005 × 10^+03^	7.618 × 10^−01^	2.049 × 10^+03^	6.476 × 10^−03^	2.025 × 10^+03^	2.741 × 10^−01^	2.056 × 10^+03^	7.356 × 10^−01^	2.144 × 10^+03^	2.077 × 10^−03^
	8	2.176 × 10^+03^	7.049 × 10^−01^	2.133 × 10^+03^	7.790 × 10^−01^	2.167 × 10^+03^	4.048 × 10^−02^	2.166 × 10^+03^	5.768 × 10^−01^	2.113 × 10^+03^	3.084 × 10^−01^	2.247 × 10^+03^	8.322 × 10^−03^
F11	2	2.035 × 10^+03^	3.069 × 10^−01^	1.968 × 10^+03^	4.495 × 10^−01^	2.003 × 10^+03^	1.254 × 10^−01^	2.074 × 10^+03^	7.945 × 10^−01^	1.990 × 10^+03^	1.179 × 10^−01^	2.199 × 10^+03^	9.718 × 10^−03^
	4	2.187 × 10^+03^	6.189 × 10^−01^	2.136 × 10^+03^	4.831 × 10^−01^	2.132 × 10^+03^	1.130 × 10^−01^	2.176 × 10^+03^	8.028 × 10^−01^	2.146 × 10^+03^	8.088 × 10^−01^	2.211 × 10^+03^	3.010 × 10^−03^
	6	2.215 × 10^+03^	7.368 × 10^−01^	2.246 × 10^+03^	5.242 × 10^−01^	2.237 × 10^+03^	7.779 × 10^−01^	2.213 × 10^+03^	3.877 × 10^−01^	2.273 × 10^+03^	3.829 × 10^−01^	2.335 × 10^+03^	2.053 × 10^−03^
	8	2.349 × 10^+03^	9.694 × 10^−01^	2.341 × 10^+03^	1.088 × 10^−01^	2.358 × 10^+03^	3.607 × 10^−01^	2.355 × 10^+03^	4.397 × 10^−01^	2.348 × 10^+03^	4.817 × 10^−01^	2.478 × 10^+03^	2.227 × 10^−03^
F12	2	2.093 × 10^+03^	1.576 × 10^−01^	2.010 × 10^+03^	4.318 × 10^−01^	1.906 × 10^+03^	7.553 × 10^−01^	1.912 × 10^+03^	7.941 × 10^−01^	1.932 × 10^+03^	2.295 × 10^−01^	2.135 × 10^+03^	2.729 × 10^−03^
	4	2.103 × 10^+03^	9.795 × 10^−01^	2.144 × 10^+03^	3.142 × 10^−01^	2.107 × 10^+03^	9.070 × 10^−01^	2.131 × 10^+03^	2.514 × 10^−02^	2.140 × 10^+03^	8.637 × 10^−01^	2.273 × 10^+03^	3.783 × 10^−04^
	6	2.217 × 10^+03^	4.049 × 10^−01^	2.223 × 10^+03^	7.166 × 10^−01^	2.294 × 10^+03^	9.043 × 10^−01^	2.266 × 10^+03^	9.975 × 10^−01^	2.204 × 10^+03^	6.749 × 10^−01^	2.348 × 10^+03^	3.577 × 10^−03^
	8	2.302 × 10^+03^	3.994 × 10^−01^	2.355 × 10^+03^	9.461 × 10^−01^	2.350 × 10^+03^	8.854 × 10^−01^	2.356 × 10^+03^	7.773 × 10^−02^	2.358 × 10^+03^	4.363 × 10^−01^	2.410 × 10^+03^	8.887 × 10^−03^
Mean Rank		3.98		4.38		4.00		3.75		3.88		1.02	
Final Rank		4		6		5		2		3		1	

**Table 4:** Wilcoxon rank sum test for fitness function values.

Image	nTH	OPBNGO vs. NDFNGO	NGO vs. NDFNGO	ANBPO vs. NDFNGO	DENGO vs. NDFNGO	IMODE vs. NDFNGO
F1	2	6.768 × 10^−10^/−	5.970 × 10^−10^/−	7.815 × 10^−10^/−	1.821 × 10^−10^/−	8.752 × 10^−10^/−
	4	1.009 × 10^−04^/−	5.269 × 10^−10^/−	9.154 × 10^−10^/−	9.663 × 10^−10^/−	2.014 × 10^−10^/−
	6	4.964 × 10^−05^/−	6.541 × 10^−06^/−	9.043 × 10^−06^/−	3.130 × 10^−06^/−	7.145 × 10^−10^/−
	8	5.496 × 10^−05^/−	8.184 × 10^−06^/−	4.362 × 10^−10^/−	2.339 × 10^−10^/+	6.732 × 10^−10^/−
F2	2	5.800 × 10^−05^/−	4.235 × 10^−06^/−	2.855 × 10^−10^/−	4.548 × 10^−10^/−	5.287 × 10^−10^/−
	4	6.651 × 10^−10^/−	8.678 × 10^−06^/−	7.210 × 10^−05^/−	1.211 × 10^−05^/−	8.546 × 10^−10^/−
	6	4.902 × 10^−10^/−	7.429 × 10^−06^/−	6.244 × 10^−05^/−	5.811 × 10^−05^/−	3.985 × 10^−07^/−
	8	7.795 × 10^−10^/−	4.103 × 10^−06^/−	6.097 × 10^−05^/−	9.278 × 10^−05^/−	3.547 × 10^−07^/−
F3	2	6.890 × 10^−04^/−	5.746 × 10^−05^/−	4.020 × 10^−05^/−	1.340 × 10^−04^/−	5.096 × 10^−07^/−
	4	5.425 × 10^−04^/−	3.041 × 10^−05^/−	3.204 × 10^−05^/−	8.372 × 10^−04^/−	6.602 × 10^−07^/−
	6	9.998 × 10^−04^/−	1.318 × 10^−05^/−	2.000 × 10^−05^/−	5.841 × 10^−04^/−	3.815 × 10^−07^/−
	8	8.336 × 10^−04^/−	4.741 × 10^−05^/−	2.463 × 10^−05^/−	6.398 × 10^−04^/−	7.777 × 10^−07^/−
F4	2	9.327 × 10^−04^/−	7.407 × 10^−05^/−	4.719 × 10^−05^/−	4.397 × 10^−04^/−	6.950 × 10^−07^/−
	4	1.576 × 10^−04^/−	1.543 × 10^−05^/−	4.274 × 10^−10^/−	8.568 × 10^−05^/−	5.593 × 10^−07^/−
	6	8.087 × 10^−04^/+	9.178 × 10^−05^/−	9.226 × 10^−10^/−	3.189 × 10^−05^/−	3.066 × 10^−07^/−
	8	1.939 × 10^−04^/−	5.563 × 10^−05^/−	8.574 × 10^−10^/−	6.928 × 10^−05^/−	7.088 × 10^−07^/−
F5	2	5.961 × 10^−04^/−	4.737 × 10^−05^/−	6.143 × 10^−10^/−	6.619 × 10^−05^/−	1.677 × 10^−07^/−
	4	2.461 × 10^−04^/−	5.283 × 10^−05^/−	6.545 × 10^−10^/−	2.468 × 10^−05^/−	5.735 × 10^−10^/−
	6	6.708 × 10^−04^/−	9.241 × 10^−05^/−	1.306 × 10^−10^/−	5.330 × 10^−05^/−	2.343 × 10^−10^/−
	8	4.392 × 10^−04^/−	5.346 × 10^−10^/−	9.048 × 10^−10^/−	4.552 × 10^−05^/−	7.910 × 10^−10^/−
F6	2	4.226 × 10^−10^/−	8.761 × 10^−10^/−	7.729 × 10^−10^/−	1.479 × 10^−10^/−	5.520 × 10^−10^/−
	4	8.601 × 10^−07^/−	5.520 × 10^−10^/−	3.985 × 10^−10^/−	6.545 × 10^−10^/−	5.664 × 10^−10^/−
	6	6.797 × 10^−10^/−	6.848 × 10^−10^/−	5.301 × 10^−10^/−	5.554 × 10^−06^/−	9.956 × 10^−10^/−
	8	5.673 × 10^−10^/−	8.398 × 10^−10^/−	6.438 × 10^−10^/−	6.184 × 10^−06^/−	8.317 × 10^−10^/−
F7	2	5.487 × 10^−10^/−	9.152 × 10^−10^/−	2.375 × 10^−07^/−	6.693 × 10^−05^/−	8.524 × 10^−10^/−
	4	9.462 × 10^−08^/−	5.946 × 10^−10^/−	2.533 × 10^−07^/−	1.262 × 10^−05^/−	8.839 × 10^−06^/−
	6	3.607 × 10^−07^/−	6.821 × 10^−10^/−	9.543 × 10^−07^/−	2.492 × 10^−05^/−	1.915 × 10^−05^/−
	8	9.173 × 10^−06^/−	3.377 × 10^−10^/−	2.815 × 10^−07^/−	9.841 × 10^−05^/−	9.577 × 10^−06^/−
F8	2	4.857 × 10^−06^/−	4.467 × 10^−10^/−	2.923 × 10^−07^/−	9.620 × 10^−05^/−	1.119 × 10^−05^/−
	4	5.545 × 10^−06^/−	5.971 × 10^−10^/−	4.307 × 10^−07^/−	6.637 × 10^−06^/−	8.165 × 10^−05^/−
	6	3.727 × 10^−06^/−	3.718 × 10^−10^/−	3.143 × 10^−07^/−	9.411 × 10^−05^/−	5.706 × 10^−05^/−
	8	5.594 × 10^−06^/−	9.423 × 10^−10^/−	4.912 × 10^−07^/−	4.912 × 10^−05^/−	6.574 × 10^−05^/−
F9	2	4.342 × 10^−06^/−	2.374 × 10^−10^/−	9.563 × 10^−07^/−	7.432 × 10^−06^/−	7.277 × 10^−05^/−
	4	8.214 × 10^−06^/−	6.600 × 10^−10^/−	6.054 × 10^−07^/−	1.705 × 10^−05^/−	6.248 × 10^−05^/−
	6	1.521 × 10^−06^/−	7.999 × 10^−10^/−	4.454 × 10^−10^/−	2.383 × 10^−06^/−	8.007 × 10^−05^/−
	8	5.334 × 10^−06^/−	2.365 × 10^−10^/−	2.270 × 10^−10^/−	9.035 × 10^−05^/−	2.421 × 10^−05^/−
F10	2	5.834 × 10^−06^/−	4.431 × 10^−10^/−	7.957 × 10^−10^/−	1.602 × 10^−05^/−	1.460 × 10^−05^/−
	4	1.972 × 10^−06^/−	2.486 × 10^−10^/−	8.996 × 10^−10^/−	4.881 × 10^−04^/−	5.905 × 10^−10^/−
	6	9.924 × 10^−10^/−	7.601 × 10^−10^/−	8.215 × 10^−10^/−	1.301 × 10^−04^/−	6.759 × 10^−10^/−
	8	4.997 × 10^−05^/−	9.604 × 10^−10^/−	4.964 × 10^−06^/−	8.388 × 10^−04^/−	5.952 × 10^−05^/−
F11	2	7.541 × 10^−05^/−	1.503 × 10^−10^/−	5.368 × 10^−07^/−	7.290 × 10^−04^/−	9.979 × 10^−05^/−
	4	4.837 × 10^−05^/−	1.839 × 10^−10^/−	7.881 × 10^−07^/−	1.769 × 10^−04^/−	7.884 × 10^−05^/−
	6	3.215 × 10^−05^/−	6.485 × 10^−10^/−	5.249 × 10^−07^/−	1.602 × 10^−04^/−	2.874 × 10^−05^/−
	8	9.189 × 10^−05^/−	7.684 × 10^−10^/−	9.614 × 10^−07^/−	1.202 × 10^−04^/−	9.669 × 10^−05^/−
F12	2	3.791 × 10^−05^/−	4.255 × 10^−10^/−	3.207 × 10^−07^/−	1.316 × 10^−04^/−	4.115 × 10^−05^/−
	4	4.003 × 10^−05^/−	4.406 × 10^−10^/−	9.963 × 10^−10^/−	3.056 × 10^−04^/−	8.660 × 10^−05^/−
	6	9.109 × 10^−10^/−	5.117 × 10^−10^/−	1.869 × 10^−10^/−	4.933 × 10^−04^/−	6.586 × 10^−05^/−
	8	4.418 × 10^−10^/−	2.650 × 10^−10^/−	6.791 × 10^−10^/−	2.754 × 10^−10^/−	6.336 × 10^−10^/−
+/^−/=^		1/47/0	0/48/0	0/48/0	1/47/0	0/48/0

**Table 5:** PSNR metrics value.

Image	nTH	OPBNGO		NGO		ANBPO		DENGO		IMODE		NDFNGO	
		Mean	Std	Mean	Std	Mean	Std	Mean	Std	Mean	Std	Mean	Std
F1	2	18.740	3.977 × 10^−03^	18.584	7.881 × 10^−03^	18.260	2.908 × 10^−03^	18.395	4.023 × 10^−03^	18.863	5.322 × 10^−03^	19.930	9.388 × 10^−03^
	4	23.087	1.199 × 10^−03^	23.874	5.939 × 10^−03^	23.669	5.717 × 10^−03^	23.896	3.082 × 10^−03^	23.573	2.457 × 10^−03^	24.483	1.262 × 10^−03^
	6	25.571	9.486 × 10^−03^	25.388	3.049 × 10^−03^	25.347	5.440 × 10^−03^	25.632	5.732 × 10^−03^	25.319	2.779 × 10^−03^	26.897	4.736 × 10^−03^
	8	26.711	9.164 × 10^−03^	26.033	3.990 × 10^−03^	26.963	3.884 × 10^−03^	26.127	6.187 × 10^−03^	26.978	5.777 × 10^−03^	26.903	9.847 × 10^−03^
F2	2	18.028	1.689 × 10^−03^	18.026	5.161 × 10^−03^	18.385	9.102 × 10^−03^	18.360	7.021 × 10^−03^	18.702	4.119 × 10^−03^	19.803	3.654 × 10^−03^
	4	23.752	8.943 × 10^−03^	23.633	7.519 × 10^−03^	23.485	8.122 × 10^−03^	23.848	2.559 × 10^−03^	23.731	3.547 × 10^−03^	24.577	2.434 × 10^−03^
	6	25.704	4.504 × 10^−03^	25.673	8.605 × 10^−03^	25.095	9.918 × 10^−03^	25.404	1.644 × 10^−03^	25.196	8.970 × 10^−03^	26.753	2.334 × 10^−03^
	8	27.036	5.091 × 10^−03^	27.659	9.706 × 10^−03^	27.757	3.058 × 10^−03^	27.119	6.987 × 10^−03^	27.540	2.464 × 10^−03^	28.444	7.874 × 10^−03^
F3	2	18.367	2.394 × 10^−03^	18.745	1.004 × 10^−03^	18.724	6.973 × 10^−03^	18.104	4.351 × 10^−03^	18.275	3.271 × 10^−03^	19.510	4.487 × 10^−03^
	4	23.964	4.988 × 10^−03^	23.297	6.722 × 10^−03^	23.952	9.500 × 10^−03^	23.192	7.664 × 10^−03^	23.986	5.542 × 10^−03^	24.519	6.265 × 10^−03^
	6	25.806	8.541 × 10^−03^	25.925	5.226 × 10^−03^	25.385	9.534 × 10^−03^	25.997	9.863 × 10^−03^	25.751	4.506 × 10^−03^	26.922	8.833 × 10^−03^
	8	27.111	8.135 × 10^−03^	27.197	8.807 × 10^−03^	27.256	6.910 × 10^−03^	27.742	1.761 × 10^−03^	27.871	3.920 × 10^−03^	28.137	8.118 × 10^−03^
F4	2	19.544	1.408 × 10^−03^	19.389	1.122 × 10^−03^	19.295	1.050 × 10^−03^	19.836	5.128 × 10^−03^	19.168	1.118 × 10^−03^	21.361	6.479 × 10^−03^
	4	24.332	2.093 × 10^−03^	24.637	6.099 × 10^−03^	24.611	2.687 × 10^−03^	24.550	9.764 × 10^−03^	24.467	9.418 × 10^−03^	26.913	2.608 × 10^−03^
	6	26.665	3.485 × 10^−03^	26.133	8.306 × 10^−03^	26.031	1.766 × 10^−03^	26.781	5.883 × 10^−03^	26.637	5.868 × 10^−03^	28.095	2.407 × 10^−03^
	8	27.287	8.737 × 10^−03^	27.659	4.683 × 10^−03^	27.037	8.229 × 10^−03^	27.210	2.982 × 10^−03^	27.634	7.117 × 10^−03^	29.470	7.463 × 10^−03^
F5	2	19.131	5.772 × 10^−03^	19.620	6.167 × 10^−03^	19.272	4.066 × 10^−03^	19.669	9.460 × 10^−03^	19.775	1.141 × 10^−03^	20.477	1.123 × 10^−03^
	4	24.819	9.830 × 10^−03^	24.433	8.877 × 10^−03^	24.787	2.436 × 10^−03^	24.229	2.065 × 10^−03^	24.021	5.482 × 10^−03^	25.328	2.462 × 10^−03^
	6	26.064	8.485 × 10^−03^	26.983	7.877 × 10^−03^	26.523	6.567 × 10^−03^	26.407	5.778 × 10^−03^	26.831	4.257 × 10^−03^	28.899	7.470 × 10^−03^
	8	27.612	5.820 × 10^−03^	27.283	7.196 × 10^−03^	27.823	3.544 × 10^−03^	27.409	9.456 × 10^−03^	27.996	2.001 × 10^−03^	29.174	8.352 × 10^−03^
F6	2	16.247	1.419 × 10^−03^	16.010	8.873 × 10^−03^	16.775	4.131 × 10^−03^	16.159	6.590 × 10^−03^	16.521	4.508 × 10^−03^	17.314	7.155 × 10^−03^
	4	23.527	9.483 × 10^−03^	23.735	5.313 × 10^−03^	23.059	2.197 × 10^−03^	23.355	6.979 × 10^−03^	23.177	9.638 × 10^−03^	24.701	2.858 × 10^−03^
	6	25.236	7.530 × 10^−03^	25.398	7.872 × 10^−03^	25.172	2.275 × 10^−03^	25.136	5.614 × 10^−03^	25.582	6.636 × 10^−03^	26.209	8.357 × 10^−03^
	8	27.870	1.563 × 10^−03^	27.941	5.529 × 10^−03^	27.486	9.678 × 10^−03^	27.133	7.038 × 10^−03^	27.540	7.210 × 10^−03^	28.067	1.177 × 10^−03^
F7	2	16.283	3.659 × 10^−03^	16.331	7.071 × 10^−03^	16.604	8.342 × 10^−03^	16.724	1.438 × 10^−03^	16.105	1.803 × 10^−03^	17.490	7.132 × 10^−03^
	4	25.974	6.356 × 10^−03^	25.938	8.980 × 10^−03^	25.583	7.020 × 10^−03^	25.918	4.344 × 10^−03^	25.782	2.150 × 10^−03^	26.634	8.617 × 10^−03^
	6	26.431	8.506 × 10^−03^	26.535	4.678 × 10^−03^	26.557	5.776 × 10^−03^	26.063	8.619 × 10^−03^	26.350	1.963 × 10^−03^	27.595	4.362 × 10^−03^
	8	27.606	9.900 × 10^−03^	27.979	9.828 × 10^−03^	27.739	8.358 × 10^−03^	27.100	1.276 × 10^−03^	27.222	9.397 × 10^−03^	28.600	5.510 × 10^−03^
F8	2	19.117	9.172 × 10^−03^	19.158	9.442 × 10^−03^	19.750	8.952 × 10^−03^	19.632	1.987 × 10^−03^	19.508	5.709 × 10^−03^	20.693	3.080 × 10^−03^
	4	23.029	6.384 × 10^−03^	23.340	7.239 × 10^−03^	23.522	1.259 × 10^−03^	23.024	7.986 × 10^−03^	23.169	6.014 × 10^−03^	24.588	9.057 × 10^−03^
	6	25.009	5.526 × 10^−03^	25.620	9.646 × 10^−03^	25.446	6.696 × 10^−03^	25.856	4.924 × 10^−03^	25.962	9.393 × 10^−03^	26.196	1.679 × 10^−03^
	8	27.804	9.456 × 10^−03^	27.458	5.785 × 10^−03^	27.033	9.006 × 10^−03^	27.270	4.701 × 10^−03^	27.208	6.189 × 10^−03^	28.786	4.989 × 10^−03^
F9	2	18.588	2.863 × 10^−03^	18.831	1.265 × 10^−03^	18.596	1.495 × 10^−03^	18.042	9.795 × 10^−03^	18.420	8.571 × 10^−03^	19.955	3.574 × 10^−03^
	4	23.845	2.057 × 10^−03^	23.020	5.619 × 10^−03^	23.033	2.687 × 10^−03^	23.043	2.759 × 10^−03^	23.318	6.995 × 10^−03^	24.136	1.885 × 10^−03^
	6	25.074	2.949 × 10^−03^	25.371	2.587 × 10^−03^	25.953	9.613 × 10^−03^	25.240	5.578 × 10^−03^	25.723	7.670 × 10^−03^	26.263	7.776 × 10^−03^
	8	26.616	4.596 × 10^−03^	26.240	9.472 × 10^−03^	26.714	2.008 × 10^−03^	26.713	4.985 × 10^−03^	26.082	4.613 × 10^−03^	27.131	1.313 × 10^−03^
F10	2	18.629	9.938 × 10^−03^	18.499	6.434 × 10^−03^	18.959	1.260 × 10^−03^	18.678	9.045 × 10^−03^	18.255	5.914 × 10^−03^	19.629	1.118 × 10^−03^
	4	23.667	4.888 × 10^−03^	23.436	2.346 × 10^−03^	23.557	7.938 × 10^−03^	23.745	5.932 × 10^−03^	23.948	6.893 × 10^−03^	24.124	9.246 × 10^−03^
	6	25.100	5.099 × 10^−03^	25.134	9.112 × 10^−03^	25.167	3.160 × 10^−03^	25.714	5.315 × 10^−03^	25.808	1.290 × 10^−03^	26.482	5.727 × 10^−03^
	8	27.046	2.120 × 10^−03^	27.635	8.370 × 10^−03^	27.773	5.928 × 10^−03^	27.577	6.364 × 10^−03^	27.239	4.581 × 10^−03^	28.069	3.847 × 10^−03^
F11	2	19.382	6.028 × 10^−03^	19.303	8.501 × 10^−03^	19.146	5.598 × 10^−03^	19.124	6.840 × 10^−03^	19.681	7.951 × 10^−03^	21.043	9.335 × 10^−03^
	4	24.645	6.346 × 10^−03^	24.005	3.209 × 10^−03^	24.306	3.539 × 10^−03^	24.395	7.568 × 10^−03^	24.885	4.317 × 10^−03^	26.187	8.201 × 10^−03^
	6	26.622	9.302 × 10^−03^	26.265	7.334 × 10^−03^	26.864	5.853 × 10^−03^	26.319	5.527 × 10^−03^	26.150	8.444 × 10^−03^	28.217	9.467 × 10^−03^
	8	27.820	9.381 × 10^−03^	27.817	3.474 × 10^−03^	27.869	7.291 × 10^−03^	27.384	8.049 × 10^−03^	27.203	7.090 × 10^−03^	29.103	2.735 × 10^−03^
F12	2	19.735	2.889 × 10^−03^	19.582	9.411 × 10^−03^	19.586	9.191 × 10^−03^	19.648	3.422 × 10^−03^	19.214	8.185 × 10^−03^	20.117	1.723 × 10^−03^
	4	24.797	6.820 × 10^−03^	24.862	2.122 × 10^−03^	24.836	7.953 × 10^−03^	24.139	5.769 × 10^−03^	24.164	9.248 × 10^−03^	25.030	2.209 × 10^−03^
	6	26.931	8.722 × 10^−03^	26.570	1.063 × 10^−03^	26.743	8.913 × 10^−03^	26.779	9.137 × 10^−03^	26.958	3.927 × 10^−03^	28.702	1.666 × 10^−03^
	8	27.383	9.086 × 10^−03^	27.695	1.478 × 10^−03^	27.965	5.328 × 10^−03^	27.658	5.517 × 10^−03^	27.886	3.828 × 10^−03^	29.366	5.208 × 10^−03^
Mean Rank		4.04		3.96		3.88		4.17		3.92		1.04	
Final Rank		5		4		2		6		3		1	

**Table 6:** Wilcoxon rank sum test for PSNR values.

Image	nTH	OPBNGO vs. NDFNGO	NGO vs. NDFNGO	ANBPO vs. NDFNGO	DENGO vs. NDFNGO	IMODE vs. NDFNGO
F1	2	6.227 × 10^−10^/−	4.000 × 10^−10^/−	6.943 × 10^−10^/−	5.476 × 10^−10^/−	8.475 × 10^−10^/−
	4	3.658 × 10^−04^/−	6.248 × 10^−10^/−	4.594 × 10^−10^/−	5.851 × 10^−10^/−	7.372 × 10^−10^/−
	6	2.456 × 10^−05^/−	1.920 × 10^−06^/−	5.586 × 10^−06^/−	8.045 × 10^−06^/−	7.409 × 10^−10^/−
	8	4.391 × 10^−05^/−	7.782 × 10^−06^/−	4.876 × 10^−10^/+	3.774 × 10^−10^/−	7.896 × 10^−10^/+
F2	2	5.189 × 10^−05^/−	4.759 × 10^−06^/−	3.046 × 10^−10^/−	4.542 × 10^−10^/−	6.647 × 10^−10^/−
	4	5.581 × 10^−10^/−	8.226 × 10^−06^/−	7.871 × 10^−05^/−	3.760 × 10^−05^/−	1.841 × 10^−10^/−
	6	5.856 × 10^−10^/−	2.737 × 10^−06^/−	3.821 × 10^−05^/−	9.252 × 10^−05^/−	2.408 × 10^−07^/−
	8	7.094 × 10^−10^/−	3.090 × 10^−06^/−	3.871 × 10^−06^/−	1.540 × 10^−05^/−	1.894 × 10^−07^/−
F3	2	2.467 × 10^−04^/−	5.733 × 10^−05^/−	1.908 × 10^−05^/−	2.592 × 10^−04^/−	2.541 × 10^−07^/−
	4	1.826 × 10^−04^/−	3.938 × 10^−06^/−	9.751 × 10^−05^/−	9.865 × 10^−04^/−	2.248 × 10^−07^/−
	6	5.581 × 10^−04^/−	5.362 × 10^−06^/−	6.444 × 10^−05^/−	3.415 × 10^−04^/−	5.575 × 10^−07^/−
	8	4.070 × 10^−04^/−	6.968 × 10^−06^/−	1.327 × 10^−05^/−	3.736 × 10^−04^/−	1.299 × 10^−07^/−
F4	2	7.626 × 10^−04^/−	5.237 × 10^−05^/−	7.128 × 10^−05^/−	1.766 × 10^−04^/−	6.819 × 10^−08^/−
	4	7.894 × 10^−04^/−	7.419 × 10^−05^/−	6.099 × 10^−10^/−	6.034 × 10^−05^/−	2.088 × 10^−07^/−
	6	4.223 × 10^−04^/−	1.137 × 10^−05^/−	6.156 × 10^−10^/−	3.999 × 10^−05^/−	6.844 × 10^−07^/−
	8	1.474 × 10^−04^/−	7.145 × 10^−05^/−	1.678 × 10^−10^/−	5.138 × 10^−05^/−	7.982 × 10^−07^/−
F5	2	1.553 × 10^−04^/−	7.577 × 10^−05^/−	2.573 × 10^−10^/−	6.225 × 10^−05^/−	6.983 × 10^−07^/−
	4	5.668 × 10^−04^/−	2.273 × 10^−05^/−	6.796 × 10^−10^/−	1.123 × 10^−05^/−	3.283 × 10^−10^/−
	6	1.653 × 10^−04^/−	6.702 × 10^−06^/−	4.466 × 10^−10^/−	4.830 × 10^−05^/−	6.802 × 10^−10^/−
	8	6.424 × 10^−04^/−	1.767 × 10^−10^/−	3.524 × 10^−10^/−	9.358 × 10^−05^/−	7.495 × 10^−10^/−
F6	2	9.336 × 10^−10^/−	9.203 × 10^−10^/−	1.562 × 10^−10^/−	4.535 × 10^−10^/−	5.522 × 10^−10^/−
	4	5.943 × 10^−07^/−	6.846 × 10^−10^/−	5.807 × 10^−10^/−	2.984 × 10^−10^/−	9.666 × 10^−10^/−
	6	3.179 × 10^−10^/−	6.919 × 10^−10^/−	8.190 × 10^−10^/−	1.348 × 10^−05^/−	5.541 × 10^−10^/−
	8	1.382 × 10^−10^/−	2.420 × 10^−10^/−	4.224 × 10^−10^/−	4.361 × 10^−05^/−	8.723 × 10^−10^/−
F7	2	2.929 × 10^−10^/−	3.919 × 10^−10^/−	8.356 × 10^−07^/−	9.864 × 10^−05^/−	7.028 × 10^−10^/−
	4	1.925 × 10^−06^/−	1.485 × 10^−10^/−	1.557 × 10^−07^/−	6.423 × 10^−05^/−	8.036 × 10^−05^/−
	6	4.635 × 10^−06^/−	1.825 × 10^−10^/−	3.525 × 10^−07^/−	1.717 × 10^−06^/−	8.616 × 10^−05^/−
	8	9.335 × 10^−06^/−	6.200 × 10^−10^/−	1.425 × 10^−07^/−	2.432 × 10^−05^/−	6.377 × 10^−05^/−
F8	2	1.656 × 10^−06^/−	4.604 × 10^−10^/−	5.185 × 10^−07^/−	7.578 × 10^−05^/−	5.151 × 10^−06^/−
	4	7.429 × 10^−06^/−	3.027 × 10^−10^/−	6.672 × 10^−07^/−	1.661 × 10^−05^/−	2.657 × 10^−05^/−
	6	7.212 × 10^−06^/−	2.392 × 10^−10^/−	3.096 × 10^−08^/−	2.404 × 10^−05^/−	2.838 × 10^−05^/−
	8	9.308 × 10^−07^/−	2.737 × 10^−10^/−	9.867 × 10^−07^/−	9.853 × 10^−05^/−	4.557 × 10^−05^/−
F9	2	9.409 × 10^−06^/−	4.030 × 10^−10^/−	7.760 × 10^−07^/−	3.073 × 10^−05^/−	8.876 × 10^−05^/−
	4	6.255 × 10^−06^/−	1.784 × 10^−10^/−	7.556 × 10^−07^/−	9.685 × 10^−05^/−	2.780 × 10^−05^/−
	6	2.068 × 10^−06^/−	6.312 × 10^−10^/−	8.135 × 10^−10^/−	6.635 × 10^−05^/−	1.755 × 10^−07^/−
	8	5.262 × 10^−07^/−	5.725 × 10^−10^/−	2.495 × 10^−10^/−	5.699 × 10^−06^/−	2.281 × 10^−05^/−
F10	2	7.615 × 10^−06^/−	8.218 × 10^−10^/−	4.479 × 10^−10^/−	1.536 × 10^−05^/−	8.488 × 10^−05^/−
	4	6.595 × 10^−06^/−	9.858 × 10^−10^/−	5.051 × 10^−10^/−	2.972 × 10^−04^/−	9.692 × 10^−10^/−
	6	7.716 × 10^−10^/−	4.751 × 10^−10^/−	1.431 × 10^−10^/−	7.720 × 10^−05^/−	9.101 × 10^−10^/−
	8	3.894 × 10^−05^/−	2.338 × 10^−10^/−	9.194 × 10^−06^/−	2.955 × 10^−04^/−	7.476 × 10^−06^/−
F11	2	2.082 × 10^−05^/−	9.971 × 10^−10^/−	3.335 × 10^−07^/−	6.329 × 10^−04^/−	5.873 × 10^−07^/−
	4	4.252 × 10^−05^/−	6.360 × 10^−10^/−	5.328 × 10^−07^/−	1.425 × 10^−04^/−	9.252 × 10^−05^/−
	6	2.781 × 10^−05^/−	3.622 × 10^−10^/−	8.685 × 10^−07^/−	7.460 × 10^−04^/−	4.629 × 10^−05^/−
	8	7.160 × 10^−05^/−	6.261 × 10^−10^/−	8.480 × 10^−07^/−	6.523 × 10^−04^/−	1.894 × 10^−05^/−
F12	2	4.590 × 10^−05^/−	6.689 × 10^−10^/−	7.575 × 10^−07^/−	7.057 × 10^−04^/−	4.799 × 10^−05^/−
	4	5.076 × 10^−05^/−	6.145 × 10^−10^/−	3.669 × 10^−10^/−	8.402 × 10^−05^/−	9.364 × 10^−05^/−
	6	3.667 × 10^−10^/−	7.226 × 10^−10^/−	4.580 × 10^−10^/−	6.273 × 10^−04^/−	6.569 × 10^−05^/−
	8	9.728 × 10^−10^/−	7.556 × 10^−10^/−	1.639 × 10^−10^/−	4.486 × 10^−10^/−	6.860 × 10^−10^/−
+/^−/=^		0/48/0	0/48/0	1/47/0	0/48/0	1/47/0

**Table 7:** SSIM metrics value.

Image	nTH	OPBNGO		NGO		ANBPO		DENGO		IMODE		NDFNGO	
		Mean	Std	Mean	Std	Mean	Std	Mean	Std	Mean	Std	Mean	Std
F1	2	0.751	7.950 × 10^−03^	0.753	3.433 × 10^−03^	0.753	1.852 × 10^−04^	0.751	1.177 × 10^−05^	0.751	3.810 × 10^−04^	0.779	1.134 × 10^−07^
	4	0.782	8.698 × 10^−03^	0.785	2.504 × 10^−03^	0.787	6.821 × 10^−03^	0.788	3.810 × 10^−05^	0.785	7.682 × 10^−04^	0.809	1.190 × 10^−07^
	6	0.823	8.404 × 10^−03^	0.824	5.478 × 10^−05^	0.823	1.746 × 10^−03^	0.825	1.839 × 10^−05^	0.828	3.382 × 10^−05^	0.847	1.095 × 10^−07^
	8	0.878	9.760 × 10^−03^	0.870	5.690 × 10^−03^	0.874	3.316 × 10^−03^	0.878	9.171 × 10^−05^	0.875	5.075 × 10^−04^	0.871	1.369 × 10^−07^
F2	2	0.789	9.689 × 10^−03^	0.783	6.396 × 10^−03^	0.785	4.980 × 10^−03^	0.788	7.292 × 10^−05^	0.783	3.794 × 10^−04^	0.808	1.584 × 10^−07^
	4	0.802	3.070 × 10^−03^	0.806	1.814 × 10^−03^	0.803	3.032 × 10^−03^	0.803	2.409 × 10^−05^	0.803	9.935 × 10^−04^	0.831	1.826 × 10^−07^
	6	0.821	2.020 × 10^−04^	0.820	2.174 × 10^−03^	0.827	4.261 × 10^−03^	0.828	8.858 × 10^−05^	0.823	2.638 × 10^−04^	0.859	1.777 × 10^−07^
	8	0.874	9.518 × 10^−05^	0.878	3.864 × 10^−03^	0.876	8.013 × 10^−03^	0.877	4.246 × 10^−05^	0.879	3.790 × 10^−04^	0.892	1.619 × 10^−07^
F3	2	0.717	8.156 × 10^−03^	0.710	1.822 × 10^−03^	0.720	6.206 × 10^−03^	0.716	1.542 × 10^−05^	0.719	4.582 × 10^−04^	0.735	1.209 × 10^−07^
	4	0.756	8.259 × 10^−03^	0.760	7.637 × 10^−04^	0.758	9.219 × 10^−03^	0.758	4.393 × 10^−05^	0.753	5.439 × 10^−04^	0.798	1.265 × 10^−07^
	6	0.803	8.630 × 10^−03^	0.803	3.231 × 10^−03^	0.810	7.398 × 10^−04^	0.808	5.386 × 10^−05^	0.808	3.395 × 10^−04^	0.836	1.154 × 10^−07^
	8	0.850	6.639 × 10^−03^	0.854	6.409 × 10^−03^	0.851	3.098 × 10^−03^	0.852	3.514 × 10^−05^	0.857	6.837 × 10^−04^	0.874	1.347 × 10^−07^
F4	2	0.732	2.788 × 10^−03^	0.737	1.524 × 10^−03^	0.736	6.008 × 10^−03^	0.734	7.786 × 10^−05^	0.739	1.627 × 10^−04^	0.755	1.140 × 10^−07^
	4	0.785	1.853 × 10^−03^	0.787	8.678 × 10^−03^	0.789	2.051 × 10^−03^	0.782	5.020 × 10^−07^	0.781	7.109 × 10^−04^	0.802	1.471 × 10^−07^
	6	0.813	7.219 × 10^−03^	0.813	3.403 × 10^−03^	0.815	4.427 × 10^−03^	0.813	9.583 × 10^−05^	0.814	3.660 × 10^−04^	0.818	1.201 × 10^−07^
	8	0.857	7.910 × 10^−03^	0.854	8.079 × 10^−03^	0.851	2.724 × 10^−03^	0.856	5.531 × 10^−05^	0.858	6.247 × 10^−04^	0.887	1.155 × 10^−07^
F5	2	0.712	8.281 × 10^−03^	0.714	1.883 × 10^−03^	0.714	7.176 × 10^−03^	0.711	8.966 × 10^−05^	0.712	4.687 × 10^−04^	0.744	1.135 × 10^−07^
	4	0.788	5.926 × 10^−03^	0.784	8.870 × 10^−03^	0.784	9.485 × 10^−03^	0.781	3.162 × 10^−05^	0.790	8.728 × 10^−04^	0.827	1.341 × 10^−07^
	6	0.817	3.165 × 10^−03^	0.815	3.926 × 10^−03^	0.819	3.331 × 10^−03^	0.816	3.877 × 10^−06^	0.814	9.229 × 10^−04^	0.853	1.898 × 10^−07^
	8	0.861	5.966 × 10^−03^	0.866	7.462 × 10^−03^	0.865	2.377 × 10^−03^	0.869	7.304 × 10^−07^	0.863	3.790 × 10^−04^	0.887	1.566 × 10^−07^
F6	2	0.697	9.118 × 10^−03^	0.690	5.181 × 10^−03^	0.693	6.915 × 10^−03^	0.694	7.982 × 10^−05^	0.691	4.393 × 10^−04^	0.721	1.122 × 10^−07^
	4	0.758	5.045 × 10^−03^	0.756	2.334 × 10^−03^	0.756	8.194 × 10^−03^	0.754	4.318 × 10^−06^	0.760	9.140 × 10^−05^	0.770	1.694 × 10^−07^
	6	0.807	2.424 × 10^−03^	0.804	2.267 × 10^−03^	0.801	2.105 × 10^−05^	0.806	6.401 × 10^−05^	0.802	2.095 × 10^−04^	0.815	1.408 × 10^−07^
	8	0.854	9.479 × 10^−03^	0.856	6.547 × 10^−03^	0.860	4.310 × 10^−03^	0.857	3.483 × 10^−05^	0.857	6.980 × 10^−04^	0.862	1.300 × 10^−07^
F7	2	0.789	7.739 × 10^−03^	0.786	1.682 × 10^−03^	0.785	8.538 × 10^−03^	0.783	2.795 × 10^−05^	0.784	9.661 × 10^−04^	0.815	1.509 × 10^−07^
	4	0.829	6.510 × 10^−03^	0.826	4.740 × 10^−03^	0.827	1.070 × 10^−03^	0.827	2.520 × 10^−05^	0.824	7.427 × 10^−05^	0.851	1.728 × 10^−07^
	6	0.853	2.337 × 10^−03^	0.860	4.041 × 10^−03^	0.852	7.489 × 10^−03^	0.856	1.460 × 10^−05^	0.856	3.945 × 10^−04^	0.881	1.136 × 10^−07^
	8	0.882	6.631 × 10^−03^	0.881	1.075 × 10^−03^	0.881	1.683 × 10^−03^	0.889	3.170 × 10^−05^	0.889	4.541 × 10^−04^	0.893	1.890 × 10^−07^
F8	2	0.796	8.905 × 10^−03^	0.794	8.621 × 10^−03^	0.797	8.167 × 10^−03^	0.798	5.150 × 10^−05^	0.794	4.166 × 10^−04^	0.815	1.618 × 10^−07^
	4	0.821	9.278 × 10^−05^	0.828	5.151 × 10^−03^	0.830	1.295 × 10^−03^	0.827	3.646 × 10^−05^	0.820	9.612 × 10^−04^	0.840	1.253 × 10^−07^
	6	0.873	5.037 × 10^−03^	0.873	2.974 × 10^−03^	0.874	5.220 × 10^−03^	0.877	1.608 × 10^−05^	0.874	9.612 × 10^−04^	0.888	1.700 × 10^−07^
	8	0.898	4.809 × 10^−03^	0.893	3.357 × 10^−03^	0.891	9.209 × 10^−03^	0.899	7.477 × 10^−05^	0.893	3.253 × 10^−04^	0.908	1.086 × 10^−07^
F9	2	0.759	3.649 × 10^−03^	0.751	5.363 × 10^−03^	0.754	4.675 × 10^−04^	0.752	7.712 × 10^−05^	0.755	2.252 × 10^−04^	0.779	1.812 × 10^−07^
	4	0.789	8.595 × 10^−03^	0.787	6.759 × 10^−03^	0.782	5.755 × 10^−03^	0.783	3.065 × 10^−05^	0.785	9.055 × 10^−04^	0.807	1.236 × 10^−07^
	6	0.826	4.735 × 10^−03^	0.824	7.296 × 10^−03^	0.824	9.369 × 10^−03^	0.821	6.452 × 10^−05^	0.826	4.373 × 10^−04^	0.844	1.804 × 10^−07^
	8	0.877	9.196 × 10^−03^	0.877	1.309 × 10^−03^	0.874	1.729 × 10^−03^	0.870	7.095 × 10^−05^	0.877	3.389 × 10^−04^	0.885	1.819 × 10^−07^
F10	2	0.781	2.626 × 10^−03^	0.782	4.398 × 10^−03^	0.789	9.497 × 10^−03^	0.787	8.817 × 10^−05^	0.784	4.307 × 10^−04^	0.796	1.318 × 10^−07^
	4	0.805	7.190 × 10^−03^	0.808	9.170 × 10^−03^	0.806	4.185 × 10^−03^	0.809	5.003 × 10^−05^	0.807	1.972 × 10^−04^	0.836	1.385 × 10^−07^
	6	0.823	9.777 × 10^−05^	0.822	3.359 × 10^−04^	0.826	7.430 × 10^−03^	0.826	3.118 × 10^−05^	0.828	2.671 × 10^−04^	0.855	1.856 × 10^−07^
	8	0.876	9.270 × 10^−03^	0.871	1.661 × 10^−03^	0.877	7.127 × 10^−03^	0.871	2.833 × 10^−05^	0.870	7.263 × 10^−04^	0.891	1.196 × 10^−07^
F11	2	0.732	7.939 × 10^−03^	0.737	9.805 × 10^−03^	0.737	7.115 × 10^−03^	0.740	7.370 × 10^−06^	0.740	3.371 × 10^−04^	0.750	1.475 × 10^−07^
	4	0.784	2.711 × 10^−03^	0.787	1.025 × 10^−03^	0.788	2.296 × 10^−03^	0.786	3.455 × 10^−06^	0.784	3.919 × 10^−04^	0.808	1.499 × 10^−07^
	6	0.818	9.556 × 10^−03^	0.815	9.880 × 10^−03^	0.811	7.018 × 10^−03^	0.815	8.838 × 10^−05^	0.810	6.729 × 10^−04^	0.843	1.112 × 10^−07^
	8	0.859	9.476 × 10^−03^	0.851	6.214 × 10^−03^	0.852	8.763 × 10^−03^	0.853	3.603 × 10^−05^	0.855	9.111 × 10^−04^	0.881	1.588 × 10^−07^
F12	2	0.713	7.254 × 10^−03^	0.714	1.814 × 10^−03^	0.714	2.438 × 10^−03^	0.714	4.095 × 10^−05^	0.711	1.410 × 10^−04^	0.744	1.327 × 10^−07^
	4	0.780	7.708 × 10^−03^	0.784	4.959 × 10^−03^	0.785	7.831 × 10^−03^	0.786	9.982 × 10^−05^	0.787	6.447 × 10^−04^	0.825	1.682 × 10^−07^
	6	0.816	2.283 × 10^−03^	0.820	7.529 × 10^−03^	0.816	3.676 × 10^−03^	0.816	7.475 × 10^−05^	0.814	6.491 × 10^−05^	0.851	1.082 × 10^−07^
	8	0.863	5.930 × 10^−03^	0.868	4.612 × 10^−03^	0.870	6.284 × 10^−03^	0.861	5.028 × 10^−05^	0.869	2.436 × 10^−04^	0.885	1.578 × 10^−07^
Mean Rank		4.19		4.23		3.73		3.81		3.96		1.08	
Final Rank		5		6		2		3		4		1	

**Table 8:** Wilcoxon rank sum test for SSIM values.

Image	nTH	OPBNGO vs. NDFNGO	NGO vs. NDFNGO	ANBPO vs. NDFNGO	DENGO vs. NDFNGO	IMODE vs. NDFNGO
F1	2	2.041 × 10^−10^/−	3.783 × 10^−10^/−	3.421 × 10^−10^/−	1.126 × 10^−10^/−	3.111 × 10^−10^/−
	4	1.795 × 10^−05^/−	4.787 × 10^−10^/−	8.773 × 10^−10^/−	8.997 × 10^−10^/−	8.592 × 10^−10^/−
	6	6.442 × 10^−06^/−	8.114 × 10^−06^/−	3.994 × 10^−06^/−	1.977 × 10^−06^/−	3.842 × 10^−10^/−
	8	1.329 × 10^−05^/+	2.006 × 10^−06^/−	6.310 × 10^−10^/+	8.049 × 10^−10^/+	6.564 × 10^−10^/+
F2	2	8.423 × 10^−05^/−	4.032 × 10^−06^/−	1.925 × 10^−10^/−	1.928 × 10^−10^/−	4.564 × 10^−10^/−
	4	8.094 × 10^−10^/−	7.453 × 10^−06^/−	3.232 × 10^−05^/−	5.324 × 10^−05^/−	5.939 × 10^−10^/−
	6	2.416 × 10^−10^/−	1.364 × 10^−06^/−	6.159 × 10^−05^/−	8.026 × 10^−05^/−	4.885 × 10^−07^/−
	8	8.867 × 10^−10^/−	9.917 × 10^−06^/−	2.134 × 10^−05^/−	8.664 × 10^−05^/−	6.742 × 10^−07^/−
F3	2	9.290 × 10^−04^/−	5.327 × 10^−05^/−	3.302 × 10^−05^/−	1.763 × 10^−04^/−	7.537 × 10^−07^/−
	4	1.521 × 10^−04^/−	6.430 × 10^−06^/−	2.926 × 10^−05^/−	3.881 × 10^−04^/−	3.075 × 10^−08^/−
	6	6.167 × 10^−04^/−	4.471 × 10^−05^/−	8.733 × 10^−05^/−	1.990 × 10^−04^/−	6.773 × 10^−07^/−
	8	2.756 × 10^−04^/−	6.044 × 10^−05^/−	1.046 × 10^−05^/−	3.762 × 10^−04^/−	8.068 × 10^−07^/−
F4	2	8.068 × 10^−04^/−	6.697 × 10^−05^/−	3.516 × 10^−05^/−	1.960 × 10^−04^/−	6.905 × 10^−07^/−
	4	2.259 × 10^−04^/−	2.660 × 10^−05^/−	5.586 × 10^−10^/−	2.136 × 10^−05^/−	5.636 × 10^−07^/−
	6	4.610 × 10^−04^/−	1.231 × 10^−05^/−	7.804 × 10^−10^/−	2.705 × 10^−05^/−	4.319 × 10^−07^/−
	8	5.570 × 10^−04^/−	6.757 × 10^−06^/−	2.075 × 10^−10^/−	1.322 × 10^−05^/−	6.860 × 10^−07^/−
F5	2	3.556 × 10^−04^/−	7.473 × 10^−06^/−	4.609 × 10^−10^/−	8.529 × 10^−05^/−	3.573 × 10^−07^/−
	4	5.580 × 10^−04^/−	8.848 × 10^−05^/−	9.811 × 10^−10^/−	8.799 × 10^−05^/−	1.482 × 10^−10^/−
	6	6.295 × 10^−04^/−	2.208 × 10^−05^/−	4.158 × 10^−10^/−	8.066 × 10^−06^/−	2.098 × 10^−10^/−
	8	3.709 × 10^−04^/−	3.900 × 10^−10^/−	6.114 × 10^−10^/−	2.783 × 10^−05^/−	2.230 × 10^−10^/−
F6	2	5.399 × 10^−10^/−	2.946 × 10^−10^/−	9.850 × 10^−10^/−	4.750 × 10^−10^/−	3.629 × 10^−10^/−
	4	7.928 × 10^−07^/−	8.631 × 10^−10^/−	7.297 × 10^−10^/−	7.315 × 10^−10^/−	1.398 × 10^−10^/−
	6	1.092 × 10^−10^/−	4.448 × 10^−10^/−	8.100 × 10^−10^/−	7.545 × 10^−06^/−	6.895 × 10^−10^/−
	8	7.968 × 10^−10^/−	3.103 × 10^−10^/−	5.139 × 10^−10^/−	9.493 × 10^−06^/−	7.622 × 10^−10^/−
F7	2	1.219 × 10^−10^/−	4.164 × 10^−10^/−	8.134 × 10^−07^/−	6.562 × 10^−05^/−	9.404 × 10^−10^/−
	4	3.528 × 10^−06^/−	1.236 × 10^−10^/−	9.807 × 10^−07^/−	7.534 × 10^−06^/−	2.574 × 10^−05^/−
	6	5.792 × 10^−06^/−	2.924 × 10^−10^/−	9.120 × 10^−07^/−	5.866 × 10^−05^/−	1.482 × 10^−05^/−
	8	6.946 × 10^−06^/−	4.305 × 10^−10^/−	8.149 × 10^−07^/−	4.446 × 10^−05^/−	9.107 × 10^−05^/−
F8	2	5.899 × 10^−06^/−	8.632 × 10^−10^/−	5.722 × 10^−07^/−	8.540 × 10^−07^/−	4.260 × 10^−05^/−
	4	1.708 × 10^−06^/−	1.496 × 10^−10^/−	4.497 × 10^−07^/−	3.133 × 10^−05^/−	6.143 × 10^−05^/−
	6	7.575 × 10^−06^/−	3.622 × 10^−10^/−	2.188 × 10^−07^/−	1.890 × 10^−05^/−	8.983 × 10^−05^/−
	8	7.488 × 10^−06^/−	8.792 × 10^−10^/−	5.277 × 10^−07^/−	6.054 × 10^−05^/−	1.746 × 10^−05^/−
F9	2	7.898 × 10^−07^/−	2.841 × 10^−10^/−	9.842 × 10^−07^/−	2.053 × 10^−05^/−	9.565 × 10^−05^/−
	4	8.630 × 10^−06^/−	3.841 × 10^−10^/−	7.581 × 10^−07^/−	6.704 × 10^−06^/−	9.730 × 10^−05^/−
	6	1.503 × 10^−06^/−	8.899 × 10^−10^/−	9.126 × 10^−10^/−	9.144 × 10^−05^/−	8.932 × 10^−05^/−
	8	5.314 × 10^−06^/−	3.261 × 10^−10^/−	4.263 × 10^−10^/−	3.656 × 10^−05^/−	5.404 × 10^−05^/−
F10	2	2.977 × 10^−06^/−	2.311 × 10^−10^/−	7.012 × 10^−10^/−	5.589 × 10^−05^/−	4.991 × 10^−06^/−
	4	3.092 × 10^−06^/−	8.917 × 10^−10^/−	3.015 × 10^−10^/−	4.973 × 10^−04^/−	2.561 × 10^−10^/−
	6	1.114 × 10^−10^/−	1.029 × 10^−10^/−	6.344 × 10^−10^/−	8.190 × 10^−04^/−	1.569 × 10^−10^/−
	8	3.339 × 10^−05^/−	1.500 × 10^−10^/−	9.551 × 10^−06^/−	5.952 × 10^−04^/−	3.903 × 10^−06^/−
F11	2	6.204 × 10^−05^/−	1.136 × 10^−10^/−	3.759 × 10^−08^/−	1.532 × 10^−04^/−	8.193 × 10^−05^/−
	4	4.641 × 10^−05^/−	5.562 × 10^−10^/−	4.955 × 10^−07^/−	2.750 × 10^−04^/−	5.610 × 10^−05^/−
	6	9.019 × 10^−05^/−	8.999 × 10^−10^/−	9.728 × 10^−07^/−	3.927 × 10^−04^/−	6.577 × 10^−05^/−
	8	5.499 × 10^−05^/−	3.666 × 10^−10^/−	3.954 × 10^−07^/−	6.627 × 10^−04^/−	8.314 × 10^−05^/−
F12	2	6.267 × 10^−05^/−	3.528 × 10^−10^/−	2.395 × 10^−07^/−	4.585 × 10^−04^/−	9.120 × 10^−05^/−
	4	9.563 × 10^−05^/−	6.784 × 10^−10^/−	3.732 × 10^−10^/−	6.673 × 10^−04^/−	8.262 × 10^−05^/−
	6	2.683 × 10^−10^/−	5.494 × 10^−10^/−	6.314 × 10^−10^/−	2.260 × 10^−04^/−	5.989 × 10^−05^/−
	8	8.553 × 10^−10^/−	9.174 × 10^−10^/−	9.590 × 10^−10^/−	5.271 × 10^−10^/−	4.052 × 10^−10^/−
+/^−/=^		1/47/0	0/48/0	1/47/0	1/47/0	1/47/0

**Table 9:** FSIM metrics value.

Image	nTH	OPBNGO		NGO		ANBPO		DENGO		IMODE		NDFNGO	
		Mean	Std	Mean	Std	Mean	Std	Mean	Std	Mean	Std	Mean	Std
F1	2	0.792	3.582 × 10^−10^	0.795	1.598 × 10^−10^	0.791	4.480 × 10^−10^	0.799	6.564 × 10^−10^	0.796	7.750 × 10^−10^	0.802	3.955 × 10^−10^
	4	0.821	6.922 × 10^−10^	0.826	1.719 × 10^−10^	0.824	4.620 × 10^−10^	0.829	3.483 × 10^−10^	0.821	4.482 × 10^−10^	0.835	4.623 × 10^−10^
	6	0.852	7.270 × 10^−10^	0.852	6.247 × 10^−10^	0.854	2.721 × 10^−10^	0.856	8.876 × 10^−10^	0.851	1.358 × 10^−10^	0.865	7.398 × 10^−10^
	8	0.875	3.212 × 10^−10^	0.870	9.669 × 10^−10^	0.872	5.288 × 10^−10^	0.873	5.569 × 10^−10^	0.872	6.637 × 10^−10^	0.874	9.897 × 10^−10^
F2	2	0.797	9.263 × 10^−10^	0.796	7.064 × 10^−10^	0.791	6.567 × 10^−10^	0.797	2.134 × 10^−10^	0.791	3.836 × 10^−10^	0.802	8.251 × 10^−10^
	4	0.828	4.873 × 10^−10^	0.821	9.297 × 10^−10^	0.823	2.879 × 10^−10^	0.830	2.077 × 10^−10^	0.826	2.129 × 10^−10^	0.846	1.863 × 10^−10^
	6	0.854	5.704 × 10^−10^	0.851	6.564 × 10^−10^	0.859	5.436 × 10^−10^	0.855	8.569 × 10^−10^	0.858	5.795 × 10^−10^	0.872	2.039 × 10^−10^
	8	0.879	6.078 × 10^−10^	0.870	1.138 × 10^−10^	0.871	1.341 × 10^−10^	0.872	2.286 × 10^−10^	0.878	4.164 × 10^−10^	0.895	1.508 × 10^−10^
F3	2	0.802	2.232 × 10^−10^	0.805	5.087 × 10^−10^	0.809	6.191 × 10^−10^	0.803	1.405 × 10^−10^	0.805	8.023 × 10^−10^	0.811	3.932 × 10^−10^
	4	0.831	1.410 × 10^−10^	0.836	3.233 × 10^−10^	0.831	6.418 × 10^−10^	0.831	4.264 × 10^−10^	0.837	7.238 × 10^−10^	0.854	8.247 × 10^−10^
	6	0.868	8.651 × 10^−10^	0.864	1.650 × 10^−10^	0.868	2.254 × 10^−10^	0.866	3.181 × 10^−10^	0.869	7.594 × 10^−10^	0.881	9.435 × 10^−10^
	8	0.879	5.077 × 10^−10^	0.877	1.274 × 10^−10^	0.878	3.715 × 10^−10^	0.879	2.945 × 10^−10^	0.877	1.768 × 10^−10^	0.896	7.914 × 10^−10^
F4	2	0.776	6.847 × 10^−10^	0.777	4.165 × 10^−10^	0.772	4.514 × 10^−10^	0.779	2.233 × 10^−10^	0.779	2.201 × 10^−10^	0.805	8.353 × 10^−10^
	4	0.805	5.118 × 10^−10^	0.806	9.995 × 10^−10^	0.804	3.952 × 10^−10^	0.803	5.371 × 10^−10^	0.801	9.500 × 10^−10^	0.827	4.223 × 10^−10^
	6	0.835	3.416 × 10^−10^	0.831	8.656 × 10^−10^	0.833	8.759 × 10^−10^	0.839	3.093 × 10^−10^	0.835	3.818 × 10^−10^	0.839	7.106 × 10^−10^
	8	0.857	8.863 × 10^−10^	0.859	3.273 × 10^−10^	0.852	4.462 × 10^−10^	0.858	8.388 × 10^−10^	0.850	6.001 × 10^−10^	0.891	3.975 × 10^−10^
F5	2	0.789	8.044 × 10^−10^	0.784	7.802 × 10^−10^	0.782	8.811 × 10^−10^	0.788	3.348 × 10^−10^	0.783	8.307 × 10^−10^	0.794	4.254 × 10^−10^
	4	0.811	8.884 × 10^−10^	0.816	5.267 × 10^−10^	0.816	1.740 × 10^−10^	0.820	3.156 × 10^−10^	0.817	7.513 × 10^−10^	0.837	5.407 × 10^−10^
	6	0.838	3.824 × 10^−10^	0.835	6.882 × 10^−10^	0.836	6.696 × 10^−10^	0.832	1.820 × 10^−10^	0.838	7.729 × 10^−10^	0.874	9.272 × 10^−10^
	8	0.855	4.741 × 10^−10^	0.854	6.363 × 10^−10^	0.851	6.961 × 10^−10^	0.850	5.972 × 10^−10^	0.857	8.366 × 10^−10^	0.893	5.496 × 10^−10^
F6	2	0.757	7.677 × 10^−10^	0.754	2.670 × 10^−10^	0.751	4.163 × 10^−10^	0.755	4.338 × 10^−10^	0.757	8.012 × 10^−10^	0.774	3.754 × 10^−10^
	4	0.791	5.936 × 10^−10^	0.790	8.439 × 10^−10^	0.796	7.908 × 10^−10^	0.795	6.918 × 10^−10^	0.793	6.394 × 10^−10^	0.817	4.901 × 10^−10^
	6	0.828	7.456 × 10^−10^	0.824	6.782 × 10^−10^	0.829	6.518 × 10^−10^	0.825	4.108 × 10^−10^	0.826	3.021 × 10^−10^	0.849	3.374 × 10^−10^
	8	0.856	3.085 × 10^−10^	0.858	8.229 × 10^−10^	0.851	2.160 × 10^−10^	0.852	3.895 × 10^−10^	0.859	4.278 × 10^−10^	0.883	9.902 × 10^−10^
F7	2	0.811	4.258 × 10^−10^	0.815	6.702 × 10^−10^	0.817	2.310 × 10^−10^	0.810	2.784 × 10^−10^	0.811	2.825 × 10^−10^	0.824	8.284 × 10^−10^
	4	0.833	6.192 × 10^−10^	0.838	1.671 × 10^−10^	0.833	2.683 × 10^−10^	0.838	1.776 × 10^−10^	0.831	1.085 × 10^−10^	0.843	3.191 × 10^−10^
	6	0.867	8.981 × 10^−10^	0.864	3.647 × 10^−10^	0.862	1.183 × 10^−10^	0.865	7.560 × 10^−10^	0.862	9.990 × 10^−10^	0.872	5.234 × 10^−10^
	8	0.878	4.767 × 10^−10^	0.875	7.128 × 10^−10^	0.871	7.024 × 10^−10^	0.871	5.229 × 10^−10^	0.876	3.522 × 10^−10^	0.893	4.809 × 10^−10^
F8	2	0.823	7.040 × 10^−10^	0.821	7.878 × 10^−10^	0.826	7.774 × 10^−10^	0.830	1.233 × 10^−10^	0.829	3.618 × 10^−10^	0.835	4.188 × 10^−10^
	4	0.849	7.075 × 10^−10^	0.840	5.639 × 10^−10^	0.846	9.738 × 10^−10^	0.849	1.285 × 10^−10^	0.846	7.590 × 10^−10^	0.872	2.083 × 10^−10^
	6	0.863	5.392 × 10^−10^	0.865	6.742 × 10^−10^	0.866	4.634 × 10^−10^	0.868	9.521 × 10^−10^	0.865	4.466 × 10^−10^	0.890	2.810 × 10^−10^
	8	0.894	9.863 × 10^−10^	0.893	8.140 × 10^−10^	0.892	3.654 × 10^−10^	0.895	6.293 × 10^−10^	0.894	4.030 × 10^−10^	0.901	8.793 × 10^−10^
F9	2	0.797	7.451 × 10^−10^	0.791	1.304 × 10^−10^	0.796	6.096 × 10^−10^	0.790	6.537 × 10^−10^	0.798	1.483 × 10^−10^	0.800	5.032 × 10^−10^
	4	0.822	3.101 × 10^−10^	0.823	4.205 × 10^−10^	0.821	9.435 × 10^−10^	0.826	7.138 × 10^−10^	0.823	5.072 × 10^−10^	0.830	2.175 × 10^−10^
	6	0.860	7.104 × 10^−10^	0.856	3.600 × 10^−10^	0.851	5.728 × 10^−10^	0.853	2.036 × 10^−10^	0.856	4.437 × 10^−10^	0.867	3.616 × 10^−10^
	8	0.872	3.185 × 10^−10^	0.871	2.127 × 10^−10^	0.878	6.274 × 10^−10^	0.878	7.036 × 10^−10^	0.870	9.024 × 10^−10^	0.885	3.868 × 10^−10^
F10	2	0.793	9.752 × 10^−10^	0.799	7.360 × 10^−10^	0.791	3.284 × 10^−10^	0.794	6.518 × 10^−10^	0.796	8.249 × 10^−10^	0.806	3.353 × 10^−10^
	4	0.828	9.910 × 10^−10^	0.828	6.575 × 10^−10^	0.828	4.683 × 10^−10^	0.823	1.406 × 10^−10^	0.829	1.447 × 10^−10^	0.847	5.214 × 10^−10^
	6	0.858	3.673 × 10^−10^	0.851	7.350 × 10^−10^	0.856	8.231 × 10^−10^	0.859	9.042 × 10^−10^	0.858	7.503 × 10^−10^	0.876	4.186 × 10^−10^
	8	0.876	2.215 × 10^−10^	0.876	9.176 × 10^−10^	0.878	2.549 × 10^−10^	0.874	1.084 × 10^−10^	0.870	7.610 × 10^−10^	0.899	1.798 × 10^−10^
F11	2	0.772	7.059 × 10^−10^	0.776	3.635 × 10^−10^	0.770	1.661 × 10^−10^	0.770	3.284 × 10^−10^	0.779	7.955 × 10^−10^	0.803	7.497 × 10^−10^
	4	0.803	6.243 × 10^−10^	0.802	4.297 × 10^−10^	0.807	8.089 × 10^−10^	0.809	4.525 × 10^−10^	0.802	8.113 × 10^−10^	0.828	7.702 × 10^−10^
	6	0.833	3.865 × 10^−10^	0.835	8.834 × 10^−10^	0.833	6.711 × 10^−10^	0.834	6.055 × 10^−10^	0.836	1.791 × 10^−10^	0.854	6.002 × 10^−10^
	8	0.852	8.366 × 10^−10^	0.860	7.668 × 10^−10^	0.857	5.390 × 10^−10^	0.859	7.654 × 10^−10^	0.852	2.106 × 10^−10^	0.893	1.863 × 10^−10^
F12	2	0.782	6.817 × 10^−10^	0.788	1.326 × 10^−10^	0.787	2.074 × 10^−10^	0.786	4.141 × 10^−10^	0.788	3.203 × 10^−10^	0.790	4.985 × 10^−10^
	4	0.816	3.454 × 10^−10^	0.813	1.127 × 10^−10^	0.814	4.790 × 10^−10^	0.814	8.003 × 10^−10^	0.817	4.417 × 10^−10^	0.838	2.051 × 10^−10^
	6	0.840	7.493 × 10^−10^	0.831	7.496 × 10^−10^	0.838	1.059 × 10^−10^	0.839	6.530 × 10^−10^	0.838	6.719 × 10^−10^	0.872	6.110 × 10^−10^
	8	0.853	6.507 × 10^−10^	0.855	7.537 × 10^−10^	0.850	8.037 × 10^−10^	0.853	7.085 × 10^−10^	0.855	7.287 × 10^−10^	0.893	8.096 × 10^−10^
Mean Rank		3.85		4.31		4.42		3.60		3.77		1.04	
Final Rank		4		5		6		2		3		1	

**Table 10:** Wilcoxon rank sum test for FSIM values.

Image	nTH	OPBNGO vs. NDFNGO	NGO vs. NDFNGO	ANBPO vs. NDFNGO	DENGO vs. NDFNGO	IMODE vs. NDFNGO
F1	2	2.175 × 10^−10^/−	8.927 × 10^−10^/−	1.273 × 10^−10^/−	3.529 × 10^−10^/−	4.455 × 10^−10^/−
	4	1.018 × 10^−05^/−	8.487 × 10^−10^/−	7.275 × 10^−10^/−	3.486 × 10^−10^/−	4.896 × 10^−10^/−
	6	9.423 × 10^−05^/−	1.820 × 10^−06^/−	9.258 × 10^−07^/−	7.982 × 10^−06^/−	2.860 × 10^−10^/−
	8	3.526 × 10^−05^/+	6.802 × 10^−06^/−	4.131 × 10^−10^/−	9.953 × 10^−10^/−	5.324 × 10^−10^/−
F2	2	5.041 × 10^−05^/−	4.583 × 10^−06^/−	4.635 × 10^−10^/−	5.420 × 10^−10^/−	2.590 × 10^−10^/−
	4	5.400 × 10^−10^/−	1.829 × 10^−06^/−	7.475 × 10^−05^/−	4.405 × 10^−05^/−	7.943 × 10^−10^/−
	6	7.524 × 10^−10^/−	4.937 × 10^−06^/−	5.517 × 10^−05^/−	5.918 × 10^−05^/−	5.963 × 10^−07^/−
	8	9.069 × 10^−10^/−	6.514 × 10^−06^/−	6.711 × 10^−05^/−	7.567 × 10^−05^/−	5.719 × 10^−09^/−
F3	2	3.339 × 10^−04^/−	9.394 × 10^−05^/−	6.789 × 10^−05^/−	6.186 × 10^−04^/−	4.781 × 10^−07^/−
	4	1.948 × 10^−04^/−	6.902 × 10^−05^/−	7.173 × 10^−05^/−	9.650 × 10^−04^/−	6.114 × 10^−07^/−
	6	7.223 × 10^−04^/−	7.070 × 10^−05^/−	3.729 × 10^−05^/−	3.315 × 10^−04^/−	8.324 × 10^−07^/−
	8	3.075 × 10^−04^/−	1.168 × 10^−05^/−	5.206 × 10^−05^/−	9.456 × 10^−04^/−	6.981 × 10^−07^/−
F4	2	7.867 × 10^−04^/−	9.564 × 10^−05^/−	9.800 × 10^−05^/−	8.744 × 10^−04^/−	6.152 × 10^−07^/−
	4	1.307 × 10^−04^/−	7.171 × 10^−05^/−	6.716 × 10^−10^/−	2.882 × 10^−05^/−	5.918 × 10^−07^/−
	6	3.866 × 10^−04^/−	6.966 × 10^−05^/−	7.032 × 10^−10^/−	6.640 × 10^−05^/+	4.244 × 10^−07^/−
	8	1.098 × 10^−04^/−	7.070 × 10^−05^/−	4.138 × 10^−10^/−	7.727 × 10^−06^/−	4.353 × 10^−07^/−
F5	2	7.694 × 10^−04^/−	4.673 × 10^−05^/−	5.971 × 10^−10^/−	8.480 × 10^−05^/−	9.401 × 10^−07^/−
	4	6.441 × 10^−04^/−	5.025 × 10^−05^/−	3.385 × 10^−10^/−	8.101 × 10^−05^/−	2.966 × 10^−10^/−
	6	6.809 × 10^−04^/−	4.661 × 10^−06^/−	4.377 × 10^−10^/−	4.610 × 10^−05^/−	9.371 × 10^−10^/−
	8	9.701 × 10^−04^/−	9.107 × 10^−10^/−	8.452 × 10^−10^/−	9.079 × 10^−05^/−	2.303 × 10^−10^/−
F6	2	8.654 × 10^−10^/−	8.417 × 10^−10^/−	2.435 × 10^−10^/−	9.868 × 10^−10^/−	6.733 × 10^−10^/−
	4	1.664 × 10^−07^/−	5.362 × 10^−10^/−	5.826 × 10^−10^/−	8.087 × 10^−10^/−	1.276 × 10^−10^/−
	6	5.839 × 10^−10^/−	5.119 × 10^−10^/−	8.437 × 10^−10^/−	8.766 × 10^−05^/−	2.175 × 10^−10^/−
	8	3.519 × 10^−10^/−	6.324 × 10^−10^/−	5.102 × 10^−10^/−	9.024 × 10^−05^/−	7.821 × 10^−10^/−
F7	2	5.858 × 10^−10^/−	6.881 × 10^−10^/−	7.920 × 10^−07^/−	2.640 × 10^−05^/−	9.232 × 10^−10^/−
	4	1.807 × 10^−06^/−	1.235 × 10^−10^/−	1.238 × 10^−07^/−	6.248 × 10^−07^/−	1.694 × 10^−05^/−
	6	7.590 × 10^−06^/−	7.815 × 10^−10^/−	1.757 × 10^−07^/−	2.171 × 10^−05^/−	7.424 × 10^−05^/−
	8	6.771 × 10^−06^/−	6.998 × 10^−10^/−	1.041 × 10^−07^/−	3.212 × 10^−05^/−	6.192 × 10^−05^/−
F8	2	8.609 × 10^−06^/−	3.800 × 10^−10^/−	1.336 × 10^−07^/−	2.178 × 10^−05^/−	2.731 × 10^−06^/−
	4	2.217 × 10^−06^/−	9.929 × 10^−10^/−	4.068 × 10^−07^/−	4.589 × 10^−05^/−	8.182 × 10^−05^/−
	6	7.326 × 10^−06^/−	7.891 × 10^−10^/−	5.728 × 10^−07^/−	4.750 × 10^−05^/−	4.922 × 10^−05^/−
	8	9.340 × 10^−06^/−	1.097 × 10^−10^/−	5.184 × 10^−07^/−	6.623 × 10^−05^/−	4.823 × 10^−06^/−
F9	2	3.666 × 10^−06^/−	1.018 × 10^−10^/−	4.891 × 10^−07^/−	5.444 × 10^−05^/−	2.355 × 10^−05^/−
	4	3.986 × 10^−06^/−	6.142 × 10^−10^/−	3.678 × 10^−07^/−	1.586 × 10^−06^/−	8.363 × 10^−05^/−
	6	1.011 × 10^−06^/−	9.665 × 10^−10^/−	1.301 × 10^−10^/−	3.634 × 10^−05^/−	1.470 × 10^−05^/−
	8	9.897 × 10^−06^/−	5.538 × 10^−10^/−	3.913 × 10^−10^/−	2.533 × 10^−07^/−	2.549 × 10^−06^/−
F10	2	3.851 × 10^−06^/−	1.356 × 10^−10^/−	8.580 × 10^−10^/−	1.547 × 10^−05^/−	5.992 × 10^−05^/−
	4	8.248 × 10^−06^/−	9.784 × 10^−10^/−	1.620 × 10^−10^/−	1.301 × 10^−05^/−	1.999 × 10^−10^/−
	6	3.320 × 10^−10^/−	7.513 × 10^−10^/−	9.400 × 10^−10^/−	7.776 × 10^−04^/−	3.307 × 10^−10^/−
	8	7.760 × 10^−05^/−	7.664 × 10^−10^/−	5.908 × 10^−06^/−	8.632 × 10^−05^/−	7.357 × 10^−05^/−
F11	2	4.532 × 10^−05^/−	9.017 × 10^−10^/−	1.392 × 10^−07^/−	8.588 × 10^−04^/−	9.569 × 10^−05^/−
	4	9.433 × 10^−05^/−	5.705 × 10^−10^/−	3.826 × 10^−07^/−	5.816 × 10^−04^/−	7.437 × 10^−05^/−
	6	1.424 × 10^−05^/−	9.376 × 10^−10^/−	1.052 × 10^−07^/−	4.073 × 10^−04^/−	5.866 × 10^−05^/−
	8	6.280 × 10^−05^/−	4.052 × 10^−10^/−	4.565 × 10^−07^/−	5.309 × 10^−04^/−	1.233 × 10^−05^/−
F12	2	1.796 × 10^−05^/−	9.273 × 10^−10^/−	8.507 × 10^−07^/−	5.327 × 10^−04^/−	7.199 × 10^−05^/−
	4	4.385 × 10^−06^/−	5.758 × 10^−10^/−	4.057 × 10^−10^/−	7.322 × 10^−05^/−	2.359 × 10^−05^/−
	6	3.647 × 10^−10^/−	7.719 × 10^−10^/−	9.230 × 10^−10^/−	4.751 × 10^−04^/−	5.726 × 10^−05^/−
	8	4.274 × 10^−10^/−	2.956 × 10^−10^/−	1.371 × 10^−10^/−	9.697 × 10^−10^/−	7.947 × 10^−10^/−
+/^−/=^		1/47/0	0/48/0	0/48/0	1/47/0	1/47/0

## Data Availability

All data from this study can be obtained from the corresponding author.
